# Age attenuates the transcriptional changes that occur with sleep in the medial prefrontal cortex

**DOI:** 10.1111/acel.13021

**Published:** 2019-09-24

**Authors:** Xiaofeng Guo, Brendan T. Keenan, Dimitra Sarantopoulou, Diane C. Lim, Jie Lian, Gregory R. Grant, Allan I. Pack

**Affiliations:** ^1^ Division of Sleep Medicine Department of Medicine University of Pennsylvania Philadelphia Pennsylvania; ^2^ Institute for Translational Medicine and Therapeutics University of Pennsylvania Philadelphia Pennsylvania; ^3^ Department of Genetics University of Pennsylvania Philadelphia Pennsylvania

**Keywords:** aging, functions of sleep, medial prefrontal cortex, next‐generation RNA sequencing, sleep, sleep deprivation

## Abstract

Sleep abnormalities are common with aging. Studies show that sleep plays important roles in brain functions, and loss of sleep is associated with increased risks for neurological diseases. Here, we used RNA sequencing to explore effects of age on transcriptome changes between sleep and sleep deprivation (SD) in medial prefrontal cortex and found that transcriptional changes with sleep are attenuated in old. In particular, old mice showed a 30% reduction in the number of genes significantly altered between sleep/wake and, in general, had smaller magnitudes of changes in differentially expressed genes compared to young mice. Gene ontology analysis revealed differential age effects on certain pathways. Compared to young mice, many of the wake‐active functions were similarly induced by SD in old mice, whereas many of the sleep‐active pathways were attenuated in old mice. We found similar magnitude of changes in synaptic homeostasis genes (*Fos*, *Arc*, and *Bdnf*) induced by SD, suggesting intact synaptic upscaling on the transcript level during extended wakefulness with aging. However, sleep‐activated processes, such as DNA repair, synaptogenesis, and axon guidance, were sensitive to the effect of aging. Old mice expressed elevated levels of immune response genes when compared to young mice, and enrichment analysis using cell‐type‐specific markers indicated upregulation of microglia and oligodendrocyte genes in old mice. Moreover, gene sets of the two cell types showed age‐specific sleep/wake regulation. Ultimately, this study enhances understanding of the transcriptional changes with sleep and aging, providing potential molecular targets for future studies of age‐related sleep abnormalities and neurological disorders.

## 1 INTRODUCTION

Multiple microarray studies have examined differences in gene expression in the brains of animals during sleep, wake, and sleep deprivation (SD; for a review, see Mackiewicz, Zimmerman, Shockley, Churchill, & Pack, 2009). These studies have used bulk tissues of different brain regions in rats, mice, and birds, and whole brain in *Drosophila*. More recently, gene expression has also been studied in specific cell types using laser capture microdissection (Nikonova et al., 2017) and translating ribosome affinity purification (Bellesi et al., 2013). All studies show differential expression of many genes. Changes are generally small (Mackiewicz et al., 2009), but conserved across species (Zimmerman, Naidoo, Raizen, & Pack, 2008).

Microarray studies have led to hypotheses on the functions of sleep. One hypothesis is synaptic homeostasis (Tononi & Cirelli, 2014), that is, synaptic strengthening during wakefulness and downscaling during sleep. Another theory is that endoplasmic reticulum (ER) stress and activation of the unfolded protein response occur during wake, and recovery occurs during sleep (Brown & Naidoo, 2010). DNA repair (Bellesi, Bushey, Chini, Tononi, & Cirelli, 2016) and macromolecular synthesis in brain (Mackiewicz et al., 2007) during sleep have also been posited. Again, functional evidence is conserved across species (Zimmerman et al., 2008).

Age‐related changes in sleep/wake are also conserved across species. Sleep fragmentation occurs in older *Drosophila* (Brown et al., 2014), mice (Wimmer et al., 2013), rats (Mendelson & Bergmann, 1999), and humans (Dijk, Duffy, & Czeisler, 2000). While multiple studies describe age‐related physiological changes of sleep, there are few that analyze age‐related molecular changes of sleep. As sleep has a crucial role in the maintenance of brain health, this question is important.

This study asks whether transcriptional changes between sleep and wake are different in old versus young mice. Analyses were performed in the medial prefrontal cortex (mPFC), a brain region that likely plays a major role in age‐related changes in sleep/wake behavior and where beta‐amyloid burden has been correlated with reduced slow‐wave activity (Mander et al., 2015). We used our previous design (Mackiewicz et al., 2007), comparing gene expression at 3, 6, 9, and 12 hr after lights‐on (7 a.m.) between sleeping mice and those kept awake by gentle handling. Unlike prior microarray studies, here we employ RNA sequencing (RNA‐Seq) to provide a more complete assessment of transcriptional changes (Ozsolak & Milos, 2011).

## 2 RESULTS

### 2.1 Behavioral state and age explain different proportions of variability

**Figure 1:**
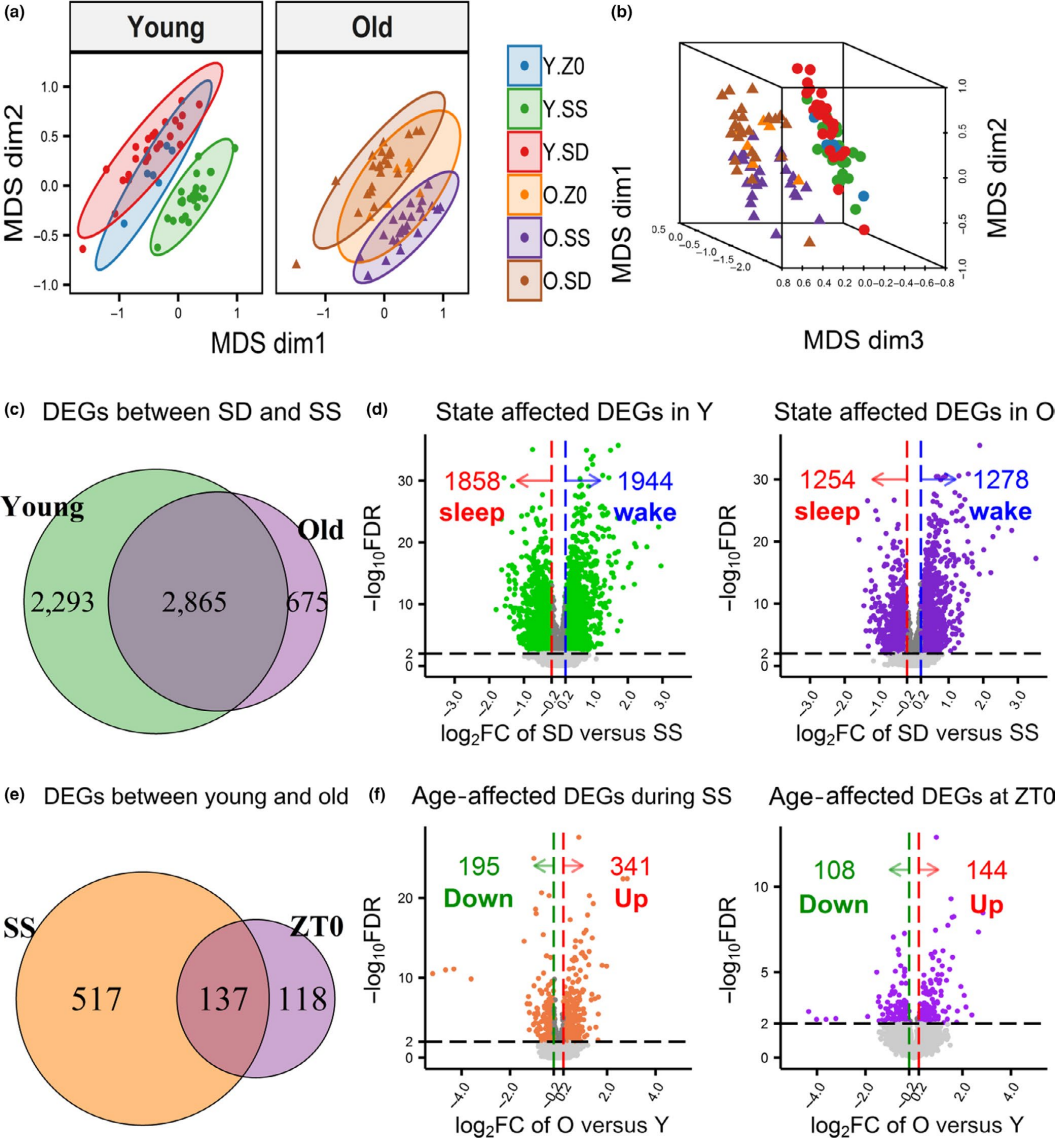
Differentially expressed genes (DEGs) between spontaneous sleep (SS) and sleep deprivation (SD) or between young and old mice. (a) Two‐dimensional plots of the multidimensional scaling (MDS) results of the young (left) and old (right) samples show SS and SD samples formed separate clusters on the first two dimensions, demonstrating that behavioral state explains the largest proportion of gene expression variability. Mice collected at ZT0 formed their own clusters between the SS and SD. (b) Three‐dimensional plot of the MDS results shows young and old samples formed separate clusters on the third dimension, illustrating age explains a smaller proportion of variability. (c) Venn diagram of the DEGs identified between SD versus SS within the young and the old mice across time points (FDR < 1%) indicate nearly 50% of genes are common. (d) Volcano plots of the −log_10_ FDR value and the log_2_ fold change (FC) between SD and SS (averaged across time points) are shown for the young (left) and old (right). DEGs with absolute log_2_ FC < 0.2 were filtered. (e) Venn diagram of the DEGs (FDR < 1%) identified in comparisons of young and old mice during SS or at ZT0 (f) Volcano plots of the −log_10_ FDR value and the log_2_ FC between old and young are plotted for the SS (left; averaged across four time points) and ZT0 (right). DEGs with absolute log_2_ FC < 0.2 filtered

Using unsupervised clustering of normalized gene expression data via multidimensional scaling (MDS) analysis, samples from different behavioral states and ages form separate clusters on a three‐dimensional space. Sleep deprivation and spontaneous sleep (SS) samples of the same age are clearly separated on the first two dimensions (Figure 1a), indicating behavioral state explains the greatest proportion of overall variability in the data. While there is some nonoverlap on the first two dimensions, young and old samples are primarily separated on the third dimension (Figure 1b), indicating age explains a smaller, but independent proportion of overall variability. Samples collected at baseline (ZT0) fall between SD and SS samples of the same age and overlap more with SD; indicating ZT0 samples more closely resemble profiles of wake.

### 2.2 Age reduces the number of differentially regulated genes between sleep/wake

We assessed differentially expressed genes (DEGs) between sleep deprived mice and time‐matched sleep controls at four time points. Using a false discovery rate (FDR) of 1%, we found 5,158 DEGs in young mice and only 3,540 DEGs in old mice. This includes 5,833 unique genes (39.8% of the 14,656 genes analyzed); 2,865 (49.1%) were differentially expressed in both ages, 2,293 (39.3%) regulated only in young mice (young‐specific), and 675 (11.6%) regulated only in old mice (old‐specific) (Figure 1c).

**Figure 2:**
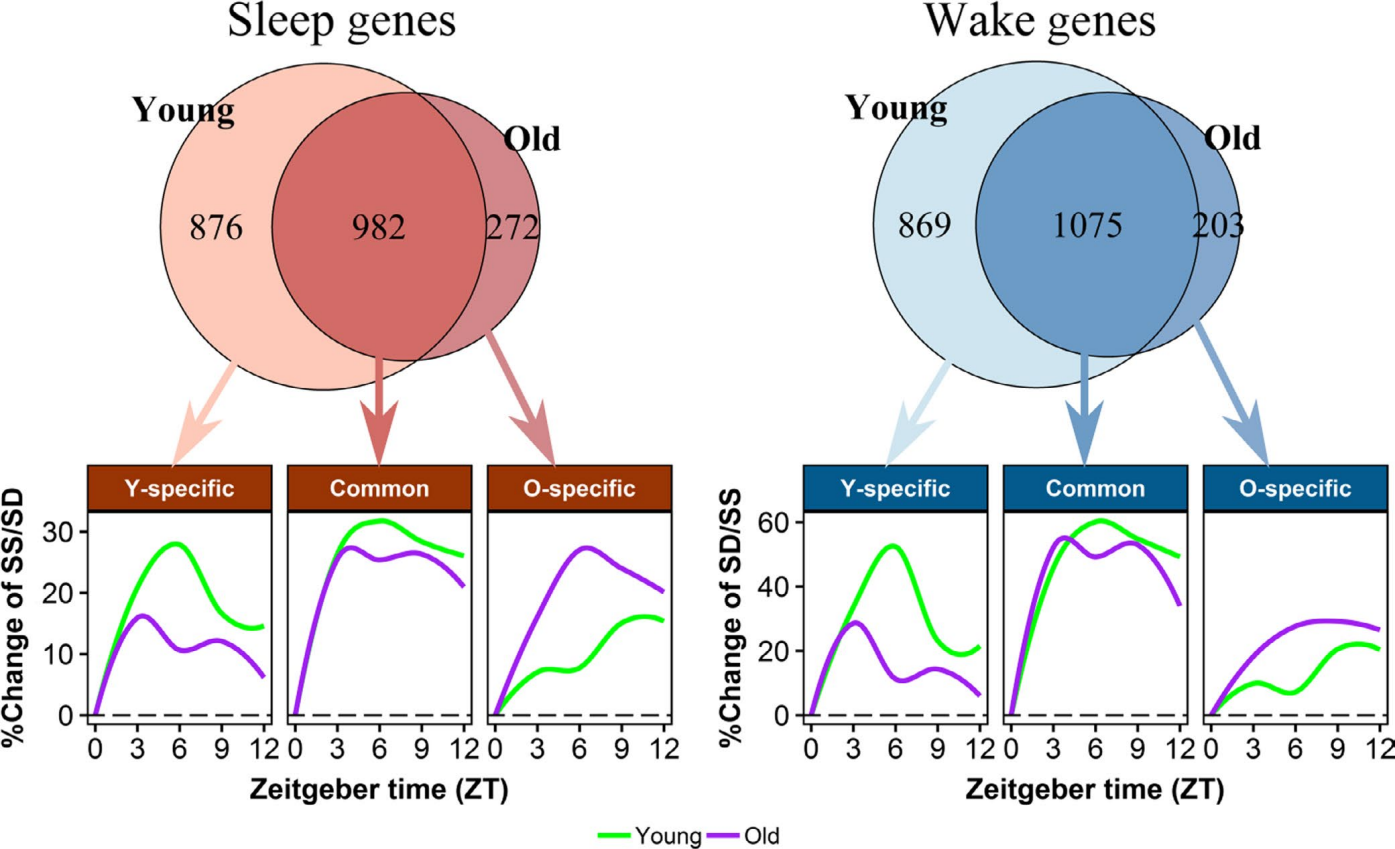
*Sleep and wake genes identified in the young and old mice*. The Venn diagrams show the number of common sleep or wake genes identified in both young and old mice, and age‐specific genes. A total of 982 sleep genes and 1,075 wake genes are common between the age‐groups. A total of 876 sleep and 869 wake genes are found only in young mice (Y‐specific), while 272 sleep and 203 wake genes are found only in old mice (O‐specific). Percentages of expression changes relative to baseline (ZT0) of each gene during SS or SD were calculated and trend lines were plotted using Loess local regression (with 95% CI) for common, young‐specific or old‐specific genes over time. Both common and young‐specific genes showed a reduction in the magnitude of change between SS and ZT0 in the old mice compared to the young. Y.SS = young mice during SS; Y.SD = young mice during SD; O.SS = old mice during SS; O.SD = old mice during SD

An absolute log_2_ fold change (FC) cutoff of 0.2 (~15% change in expression) was applied to all DEGs to remove genes with small FCs (averaged over the time points). This resulted in 3,802 and 2,532 DEGs for young and old mice, respectively. Nearly equal numbers of genes were upregulated by SD (wake genes) or upregulated during SS (sleep genes) (Figure 1d). While ~50% of genes were common to both ages, young mice had nearly 4‐times more age‐specific DEGs than old mice (Figure 2; Venn diagrams). Application of different FC cutoffs did not alter these proportions (Figure S1). Table S1 contains detailed DEG results in young and old mice.

### 2.3 Effect of age on sleep/wake genes is driven by a reduction in the magnitude of change

**Figure 3:**
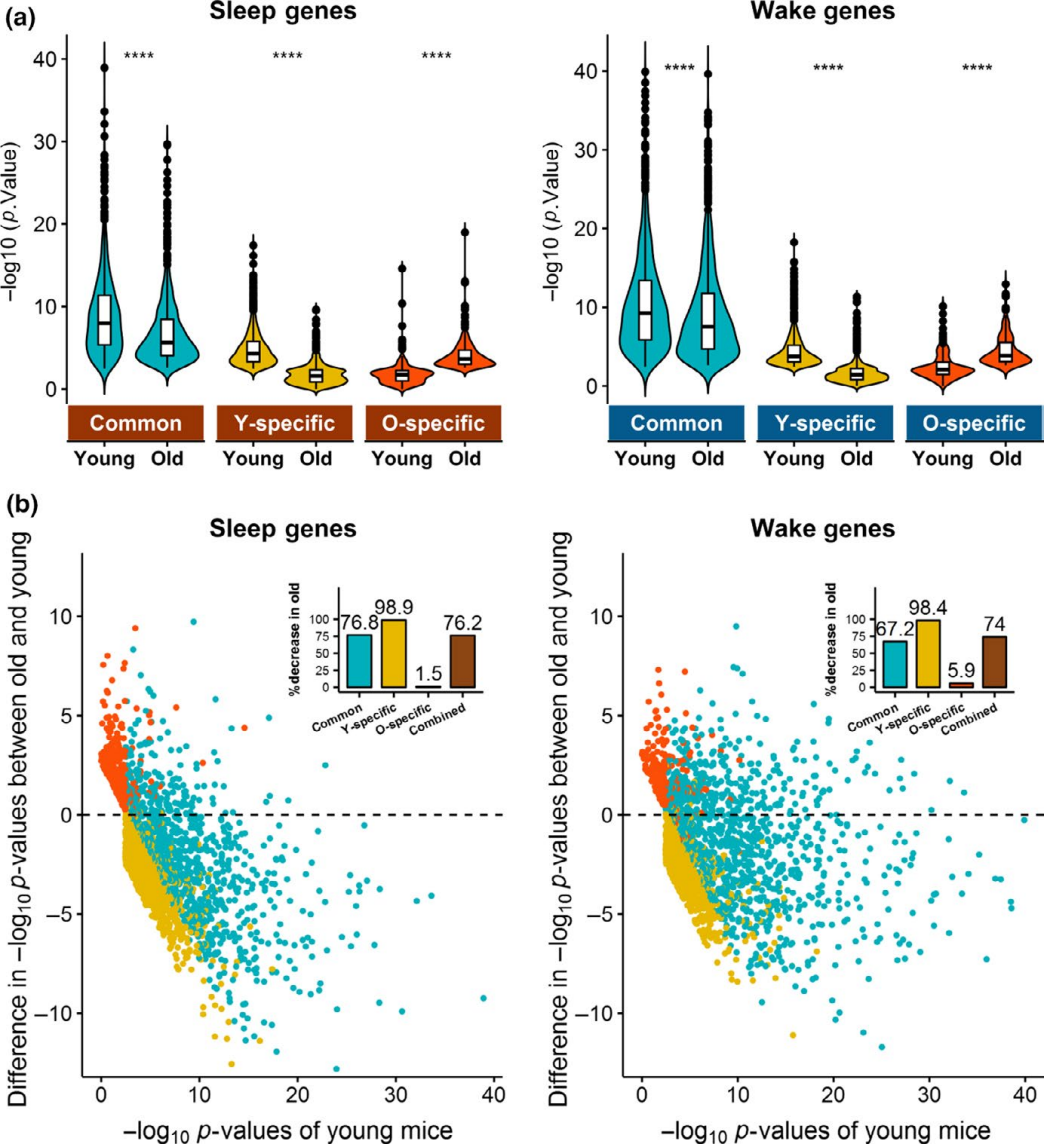
Comparisons of *p*‐values and fold changes (FCs) of sleep and wake genes between old mice and young mice. (a) –log_10_ *p* from the SD versus SS contrast was compared between young and old mice for the common or age‐specific sleep (left) and wake (right) genes. Significantly decreased –log_10_ *p* were observed in old mice in both young‐specific and common genes, which represent 87.2% of the sleep genes and 90.5% of the wake genes tested. The remaining old‐specific genes had significantly increased –log_10_P in old mice compared to young. ****: *p* < 2e−16. (b) Scatter plots of the differences in absolute values of log_2_ FC of individual sleep (left) and wake (right) genes between old and young mice. Positive differences indicate greater FC in old, and negative differences indicate greater FC in young. Inset bar plots show the percentage of common and age‐specific genes with decreased FC in old. Over 98% of the young‐specific genes, 76.2% of common sleep genes, and 65.8% of common wake genes have decreased FC in old, whereas over 97% of old‐specific genes have increased FC in old. The small percentages of age‐specific genes showing increased significance in the opposite age‐group are likely due to the applied FC cutoff that allocates similarly significant genes into age‐specific groups. Overall, 76.0% of sleep genes and 73.1% of wake genes have reduced FC in old mice

We observed a clear reduction in the number of differentially regulated sleep and wake genes in old mice. Supporting this observation, old mice had significantly decreased –log_10_ *p*‐values compared to young mice, not only in young‐specific DEGs, but, more importantly, also in common DEGs (*p* < 2e−16; Figure 3a). This reduced significance may result from increased variability in gene expression and/or reduced magnitude of change between sleep and wake in old mice. No overall trend of increased variability in old mice was found when examining the coefficient of variation (CV) across conditions (Figure S2). On the other hand, 76.1% of sleep genes and 73.1% of wake genes showed lower magnitude of FC between SD and SS in old compared to young mice (Figure 3b), exactly mirroring the percentages of genes with lower *p*‐values in old mice (76.2% and 74.0% of sleep and wake genes, respectively; Figure S3). Moreover, gene‐specific differences in FC and *p*‐values between ages were significantly correlated (Figure S4).

To further support that reduction in the magnitude of change is driving the age‐related reduction in number of DEGs, we plotted FCs between SS and SD for sleep genes and between SD and SS for wake genes, respectively, using loess regression lines and 95% confidence intervals (Figure 2). Common DEGs had similar trends over time but, showed slightly reduced magnitude of changes in the old mice compared to the young. As expected, DEGs unique to young mice had diminished changes in old mice, and DEGs unique to old mice had diminished changes in young mice, albeit similar trends of changes were observed between the two ages. Furthermore, temporal plots of gene expression over time during SS and SD (relative to ZT0) showed that age differences in majority of the genes (common and young‐specific DEGs) were driven by reduced expression changes during SS rather than during SD in old mice compared to the young (Figure S5).

### 2.4 Twenty‐one sleep/wake genes showed significant modification by age in formal interaction tests

To identify genes with significant age‐related differences in sleep and wake regulation, we tested for interaction between age‐group and behavioral state among the 2,130 sleep genes and 2,147 wake genes. Despite the lower power of interaction tests, 21 genes (12 sleep and 9 wake) showed significant interaction at an FDR < 5% (Figure S6).

Of the 12 sleep genes, 9 had greater changes in young mice, including 8 young‐specific genes (*E130307A14Rik*, *Gm15513*, *Pclo*, *Reln*, *Slc22a3*, *Epb4*.*1l5*, *Herc1*, and *Meig1*) and 1 common gene (*Slit2*). The remaining 3 genes were old‐specific (*Igf2*, *Cd36*, and *Svep1*). These 12 sleep genes are involved in various functions; the most overrepresented include *positive regulation of peptidyl‐tyrosine phosphorylation* (GO:0050731; *p = *.0027), *cell adhesion* (GO:0007155; *p = *.0060), and *protein localization to synapse* (GO:0035418; *p = *.0162) (Table S2). Of the 9 wake genes, 4 are young‐specific (*Rreb1*, *Jam3*, *Ccdc163*, and *Tuft1*), 4 are common (*Mll1*, *Mdn1*, *Mirg*, and *Morc2b*), and 1 is old‐specific (*Syne1*). No biological function was enriched among these wake genes.

### 2.5 Sleep upregulates many age‐specific functions in young and old mice

As interaction tests are underpowered, we further examined age differences in the common and age‐specific DEGs at the pathway level using functional clustering analyses. Eleven functions were enriched among sleep genes, including three among the common DEGs, five among the young‐specific DEGs and three among the old‐specific DEGs. Table 1 (top) lists names and example genes of the enriched clusters. Table S3 includes full lists of function annotation terms and genes in each cluster (and unclustered terms with *p < *.05).

**Table 1:** Enriched functions among sleep and wake genes from young and/or old mice

Gene list	Function clusters	ES^a^	*N* terms^b^	*N* genes^c^	Example genes
Enriched functions among sleep genes					
Common	DNA repair	2.05	9	41	*Exo1*, *Brca2*, *Rad50*, *Pole*, *Casp3*
	Signal/transmembrane	1.77	5	322	*Gabra4*, *Grin3a*, *Clcc1*, *Slc13a5*
	G‐protein‐coupled receptor	1.77	3	29	*Gpr12*, *Gpr21*, *Gpr25*, *Gpr165*
Young‐specific	Cell adhesion	3.98	4	75	*Cdh7*, *Pcdh10*, *Reln*, *Nrxn3*
	Axon guidance	2.33	5	32	*Met*, *Mapk1*, *Ppp3ca*, *Ppp3cb*
	Signal/transmembrane	2.22	5	287	*Abca1*, *Gpr22*, *Slitrk2*, *Slc22a3*
	Kinase activity	1.63	3	4	*Cmpk1*, *Cmpk2*, *Ak8*, *Nme5*
	Chloride channel activity	1.44	4	9	*Gabrb2*, *Gabrg2*, *Clcn3*, *Slc26a4*
Old‐specific	Collagen/glycoprotein	2.70	6	85	*Cdhr3*, *Col14a1*, *Col5a1*, *Mmp11*
	Transmembrane	2.43	5	115	*Cdhr3*, *Cacna2d4*, *Slc24a4*, *Slc9a3*
	Calcium transport	1.74	7	30	*Cdhr3*, *Cacna2d4*, *Mmp11*, *Slc9a3*
Enriched functions among wake genes					
Common	ER stress	2.77	10	57	*Hspb1*, *Hspa5*, *Dnajb5*, *Pdia4, Xbp1*
	Regulation of transcription	2.50	20	415	*Egr1*, *Fos*, *Fosb*, *Junb*, *Crem*
	Response to VEGF	2.34	3	10	*Vegfa*, *Egr3*, *Srf*, *Xbp1*
	Dephosphorylation	2.28	8	23	*Dusp1*, *Dusp4*, *Dusp5*, *Ptpn1*
	Rhythmic process	2.17	7	34	*Arntl*, *Per1*, *Per2*, *Crem*, *Sik1*
	FoxO signaling	2.16	4	34	*Cdkn1a*, *Ccnb1*, *CCnd3*
	Response to cAMP	2.15	4	32	*Crem*, *Sik1*, *Fos*, *Egr1*
	MAPK signaling	2.14	13	92	*Fos*, *Hspb1*, *Cdkn1a*
	Kinase activity	2.12	10	149	*Sik1*, *Cdkn1a*, *Ccnb1*
	Cytokine activity	1.75	6	34	*Crh*, *Scg2*, *Il12a*, *Cx3cl1*
	Dioxygenase activity	1.74	4	18	*Scd4*, *P4ha1*, *Ptgs2*, *Tet3*
	Fat cell differentiation	1.70	3	15	*Egr2*, *Nr4a1*, *Nr4a2*, *Nr4a3*
Young‐specific	Actin binding	2.15	4	42	*Crocc*, *Tns1, Fscn1, Fhod1*
	GTPase activation	1.52	3	26	*Arhgef19, Arhgap4, Arhgef1*
Old‐specific	Lipid transport	1.92	4	9	*Star*, *Plekha3*, *Plekha8*, *Snx16*
	Regulation of transcription	1.58	3	70	*Atf3*, *Ppargc1b, Zfp568*
	Negative regulation of T‐cell receptor signaling pathway	1.50	4	5	*Ptpn22*, *Dusp3*, *Dusp7*, *Ephx2*

a Enrichment score (equivalent to –log_10_‐transformed geometric mean of the *p*‐values of the included terms)

b Number of functional terms in each functional cluster

c Number of unique genes included in each functional cluster.

**Figure 4:**
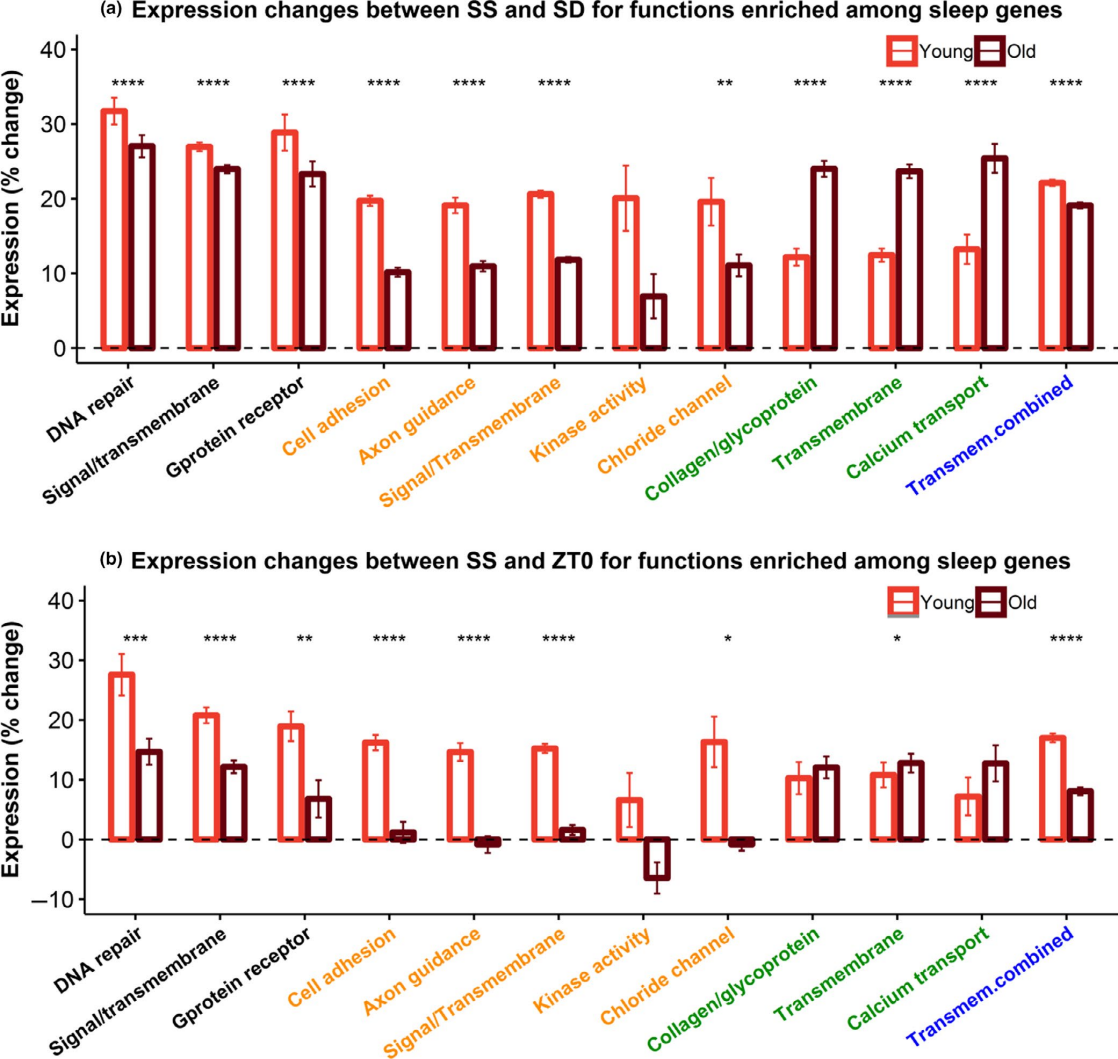
Relative expression changes (a) between SD and SS or (b) between SS and ZT0, for genes involved in the functional clusters enriched among sleep genes. Mean expression changes of the genes involved in each function are compared between young (red) and old (brown) mice. Colors on the *x*‐axis indicate if the functions are enriched among the common (black), young‐specific (orange), or old‐specific (green) sleep genes. A combined function colored in blue (“*Transmem*.*combine”*) contains genes from the three transmembrane functions enriched from common, young‐specific, and old‐specific genes. *: *p* < .05; **: *p* < .01; ***: *p* < .001; ****: *p* < .0001

To compare magnitude of changes between the two ages on the pathway level, log_2_FC of the genes involved in each enriched function was compared between young and old mice. Young animals showed greater increases in expression during SS [compared to both SD (Figure 4a) and ZT0 (Figure 4b)] in all three common functions (*DNA repair*, *Signal/transmembrane*, and *Gprotein receptor*) as well as all five young‐specific functions (*Cell Adhesion, Axon Guidance, Signal/transmembrane, Kinase activity*, and *Chloride channel activity*). Medium to large expression differences (effect sizes [Cohen's *d*] between 0.42 and 2.04) were found between young and old mice for all pathways except *Signal/transmembrane* (Table S4). As expected, old animals showed larger FC between sleep and wake in functions enriched among the old‐specific DEGs (*Collagen/glycoprotein*, *Transmembrane,* and *Calcium transport*). Interestingly, expression differences in the old‐specific pathways appear driven by greater decrease in SD (for SS vs. SD, all 3 functions reached *p < *.0001 with Cohen's *d* > 1), rather than increase in SS (for SS vs. ZT0, *p < *.05 for *Transmembrane* only and all Cohen's *d* < 0.35; Table S4).

### 2.6 Wake upregulates many common functions in young and old mice

Seventeen functional clusters were enriched in wake genes, 12 among common DEGs, 2 among young‐specific DEGs, and 3 among old‐specific DEGs. Details are listed in Table 1 (bottom) and Table S3.

**Figure 5:**
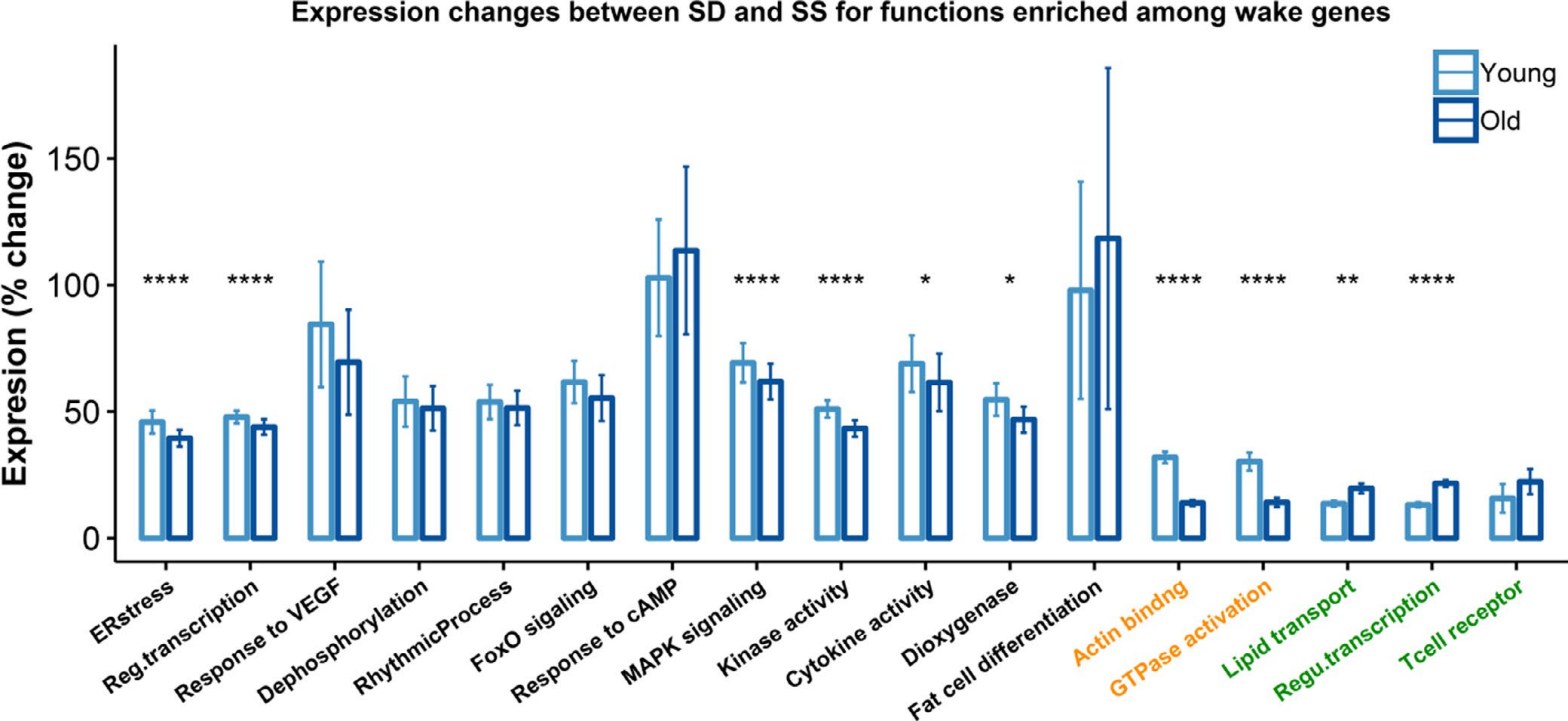
Relative expression changes (%) between SD and SS for genes involved in the functional clusters enriched among the wake genes. Mean expression changes of the genes in each function are compared between young (light blue) and old (dark blue) mice. Colors on the *x*‐axis indicate if the functions are enriched among the common (black), young‐specific (orange), or old‐specific (green) wake genes. *: *p < *.05; **: *p < *.01; ***: *p < *.001; ****: *p < *.0001

Expression changes on the pathway level were also compared between young and old mice; results are shown in Figure 5 and Table S5. Clusters enriched among young‐specific DEGs (*Actin binding* and *GTPase activation*) showed significantly greater expression increase in SD in young mice (*p* < 1e−8; Cohen's *d* > 1). Similarly, functions enriched among old‐specific DEGs showed either significant (*Lipid transport* and *Regulation of transcription*; *p < *.004; Cohen's *d* > 1) or moderate (*Negative regulation of T‐cell receptor signaling pathway*; *p = *.063; Cohen's *d* = 0.6) increase in SD in old mice. However, unlike the common sleep functions, all 12 common wake functions showed small differences between young and old mice (Cohen's *d* ≤ 0.31). While effects were small, the four functions with most genes (*n* > 50) showed highly significantly increased expression changes in young mice compared to old (*ER stress* [*p* = 8e−5], *regulation of transcription* [*p* = 2e−13], *MAPK signaling* [*p* = 1e−5], and *Kinase activity* [*p* = 2e−12]) (Table S5).

### 2.7 Immune systems upregulated and chemical synaptic transmission and nervous system development downregulated in old mice

We next focused on the effect of age in the undisturbed conditions, that is, SS and ZT0. As suggested by the MDS plot (Figure 1a,b), we found fewer DEGs between age‐groups. Precisely, 654 genes during sleep and 255 genes at ZT0 were differentially expressed between young and old mice (FDR < 1%); 137 (17.7%) were common between SS and ZT0 (Figure 1e). When applying the log_2_FC cutoff (absolute value of 0.2), 536 DEGs during sleep and 252 DEGs at ZT0 remained. More genes were found upregulated with age (341 during SS and 144 at ZT0) than downregulated with age (195 during SS and 108 at ZT0) (Figure 1f); 90 upregulated and 36 downregulated genes are common to SS and ZT0 (Figure S7). Table S1 contains detailed results of the differential gene expression analysis between young and old mice.

Multiple immune pathways are enriched from genes upregulated both during SS and at ZT0 in old mice, including *antigen processing/presentation*, *inflammatory response*, and *immune system process*. The *homophilic cell adhesion* pathway is also induced both during sleep and at ZT0 in old mice. During sleep, a few additional pathways were enriched among age upregulated genes, including *lipid metabolic process, positive regulation of myelination, apoptotic process,* and *negative regulation of neuron development*. See Table 2 for lists of individual genes in these pathways.

**Table 2:** Top enriched pathways among significantly regulated genes in old mice compared to the young mice during sleep and at ZT0

Time	Biological function term		Count	*p*	Genes
Top pathways upregulated in old mice					
SS	GO:0002474 ~ antigen processing and presentation of peptide antigen via MHC class I		9	6.4E−08	*H2‐Q6*, *H2‐Q7*, *H2‐D1*, *H2‐K1*, *Mr1*, *H2‐Gs10*, *H2‐M3*, *H2‐Bl*, *H2‐T22*
	GO:0006629 ~ lipid metabolic process		21	3.6E−05	*C3*, *Cyp39a1*, *Mboat1*, *Apod*, *Slc27a2*, *Alox12b*, *Ldlr*
	GO:0007156 ~ homophilic cell adhesion via plasma membrane adhesion molecules		11	2.4E−04	*Pcdhb2*, *Pcdhb9*, *Pcdhb6*, *Pcdhb8*, *Pcdhb3*, *Pcdhb14*, *Pvrl4*
	GO:0002376 ~ immune system process		17	3.4E−04	*Ly9*, *H2‐Q7*, *C3*, *Oas2*, *H2‐DMb1*, *Icosl H2‐K1*, *Naip5*, *Naip2*
	GO:0031643 ~ positive regulation of myelination		4	1.5E−03	*Ngfr*, *Gm98*, *Pard3*, *Trf*
	GO:0006915 ~ apoptotic process		20	1.5E−03	Casp1, Casp12, Ngfr, Tnfrsf9, Adamtsl4, Lsp1, Irak3
	GO:0010977 ~ negative regulation of neuron projection development		6	3.2E−03	*H2‐D1*, *Ngfr*, *Pmp22*, *Gfap*, *Efemp1*, *H2‐K1*
	GO:0006954 ~ inflammatory response		13	7.8E−03	*C4b*, *C3*, *Clec7a*, *Ngfr*, *Tnfrsf9*, *Agtr2*, *Naip2*, *Naip5*
ZT0	GO:0007156 ~ homophilic cell adhesion via plasma membrane adhesion molecules		11	1.2E−07	*Pcdhb2*, *Pcdhb9*, *Pcdhb6*, *Pcdhb8*, *Pcdhb3*, *Pcdhb14*, *Pcdhb4*, *Pcdhb13*
	GO:0006954 ~ inflammatory response		11	9.2E−05	*C4b*, *Clec7a*, *Agtr2*, *Ccr2*, *Parp4*, *Il1a*, *Cd180*, *Tnfrsf11b*
	GO:0045087 ~ innate immune response		11	3.1E−04	*Ly9*, *C4b*, *Clec7a*, *Cd180*, *Zc3hav1*, *C1ra*, *Cd84*, *Ifih1*
	GO:0002376 ~ immune system process		9	3.8E−03	*Ly9*, *Cd180*, *Zc3hav1*, *C1ra*, *Cd84*, *Ifih1*, *Mr1*, *Irgm1*
Top pathways downregulated in old mice					
SS		GO:0007165 ~ signal transduction	24	3.6E−04	*Gpr26*, *Gpr125*, *Gpr17*, *Gpr88*, *Npbwr1*, *Syde2*, *Rxfp2*, *Opn3*
		GO:0007626 ~ locomotory behavior	6	2.2E−03	*Penk*, *Aldh1a3*, *Calb1*, *Klhl1*, *Gpr88*, *Oprk1*
		GO:0007268 ~ chemical synaptic transmission	7	3.5E−03	Met, Npbwr1, Penk, Htr2a, Snca, Oprk1 Kcnmb4
		GO:0007399 ~ nervous system development	9	0.015	Nrsn1, Gfra2, Grip1, Nrn1, Pcdh18, Slit2, Itm2a, Sema3d, Dpysl3
ZT0		GO:0045893 ~ positive regulation of transcription DNA‐templated	11	8.2E−04	Egr2, Nr4a1, Sox4, Sox8, Npas4, Hdac1, Map2k3, Klf2, Usp21, Wnt4, Acvr1,
		GO:0071850 ~ mitotic cell cycle arrest	3	2.3E−03	Dusp1, Cdkn1a, Gadd45a
		GO:0043066 ~ negative regulation of apoptotic process	10	2.7E−03	Cdkn1a, Ccnd2, Dusp1, Raf1, Btg2, Hdac1, Plk3, Cyr61, Sox8, Siah2
		GO:0048168 ~ regulation of neuronal synaptic plasticity	3	7.4E−03	Egr2, Ephb2, Arc

Among genes downregulated in old mice during sleep, *signal transduction*, *locomotory behavior*, *chemical synaptic transmission*, and *nervous system development* are the top enriched functions (Table 2 and Table S6). At ZT0, *positive regulation of transcription, mitotic cell cycle arrest*, *negative regulation of apoptotic process*, and *regulation of neuronal synaptic plasticity* are enriched among genes downregulated with age.

### 2.8 Circadian core clock genes are affected by behavioral state and aging

Since circadian‐related functions were enriched by both behavior and age, we specifically examined how the 12 circadian core clock genes (*Bmal1/Arntl, Clock, Per1, Per2, Per3, Cry1, Cry2, Nr1d1, Nr1d2, Dbp, Tef,* and *Hif*) were affected by sleep/wake and aging.

In young mice, significantly altered expression between SD and SS was observed for all genes (FDR < 1%), except for a trending effect in *Nr1d1* (FDR *q*‐value [*q*]=0.028) and nonsignificant effect for *Per3* (Table S7). In comparison, only five genes (*Arntl*, *Per1*, *Per2*, *Dbp*, and *Tef*) were significantly altered by SD in old mice. With respect to the effects of age, *Cry1* was highly elevated in old mice during sleep (*q* = 9.3e−5). *Arntl*, *Per1*, *Per2*, and *Per3* also showed moderate changes between young and old mice at ZT0 or during SS (*q* = 0.01–0.05). *Cry1*, *Per1* and *Per2* showed reduced change during SS relative to ZT0 in old compared to young mice (Figure S8), suggesting a decline of circadian cycling in these core clock genes in old mice.

### 2.9 Synaptic homeostasis and synaptogenesis regulations between sleep and wake

We found multiple synaptic transmission‐ and synaptic plasticity‐related pathways regulated by behavior or age. Thus, we examined how known genes involved in synaptic homeostasis and synaptogenesis were affected in our data (Table S8 and Figure S9).

As many transcription factors (TFs) differentially regulated by sleep and wake are key factors of neuronal activity and synaptic plasticity (Alberini, 2009), we directly examined five – *Fos*, *Fosb*, *Egr1*, *Egr2*, and *Arc*. All were highly induced by SD in both ages (*q* ≤ 6.4e−17), with similar magnitude of change in young (2.6‐ to 7.7‐fold increase) and old (2.8‐ to 11.5‐fold increase) mice. *Bdnf* and its receptor *Ntrk2* (or *TrkB*) were also upregulated by SD in both young and old mice (Table S8).

Besides the molecular factors affecting neuronal activity, presynaptic and postsynaptic mechanisms also influence synaptic plasticity (Ho, Lee, & Martin, 2011). At presynaptic sites, genes playing key roles in synaptic assembly and neurotransmitter release were differentially regulated by sleep and wake, including *Pclo*, *Syn1* and *Syn2*, *Nrxn1* and *Nrxn3*, *Slit2*, and *Reln*. All 7 genes had significantly induced expressions during SS versus SD in young mice (9%–27% increase), while changes were reduced or nonsignificant in old mice (0%–13% increase; Table S8). At postsynaptic sites, key genes (*Gria1* and *Grin2a*) were not differentially regulated, as indicated in previous studies (Vyazovskiy, Cirelli, Pfister‐Genskow, Faraguna, & Tononi, 2008). However, other members and regulators of the glutamate receptors (*Gria4*, *Grin3a*, *Cacna1b*, *Cacna2d1*, and *Nlng1*) were significantly induced during SS compared to SD in young mice, and changes in old mice were either reduced or abolished (Table S8).

### 2.10 Enrichment of cell‐type‐specific genes revealed upregulated microglia genes in old mice

Since we obtained mPFC samples using microdissection, expression changes may come from different cell types. Using available cell‐type‐specific genes in literature from single‐cell RNA‐Seq or microarray data from isolated cells (Table S9), we tested for enrichment of specific cell‐type genes affected by behavior or age.

**Figure 6:**
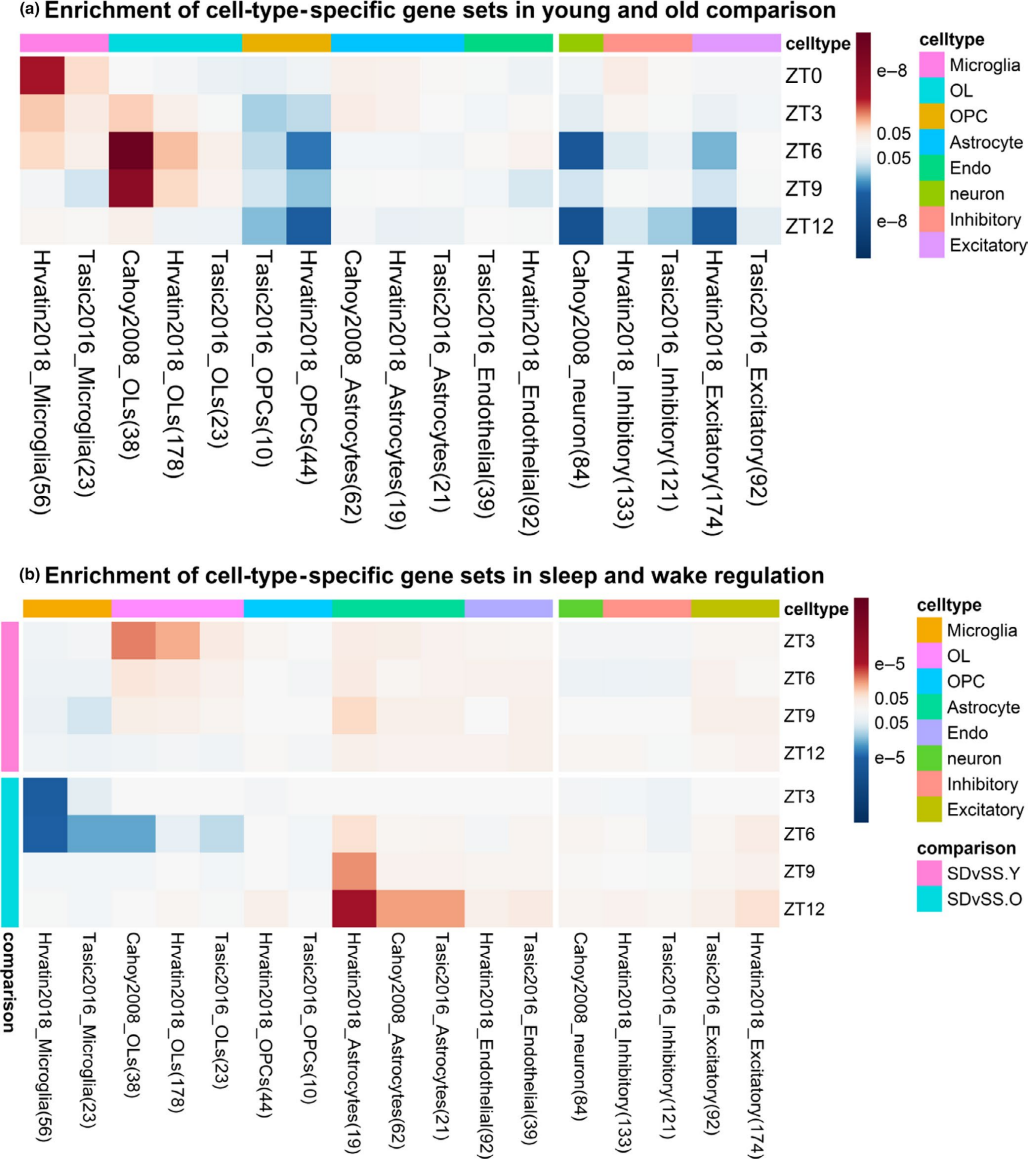
Heat map of adjusted *p*‐values for enriched cell‐type‐specific gene sets. Columns are arranged by the cell types. OL = oligodendrocyte; OPC = oligodendrocyte precursor cell. Cell colors indicate upregulation (red) and downregulation (blue), either in old for age comparisons or in SD for SD vs SS comparisons. (a) Old versus young at ZT0 and during SS; (b) SD versus SS of the young and old mice across time points

Comparing young and old mice (Figure 6a), microglia genes were significantly upregulated in old mice at ZT0 and during SS. This agrees with our finding of enriched *immune and inflammation* pathways in old mice. During sleep, significant upregulation of oligodendrocytes genes was found in old compared to young mice, which agrees with enrichment of the *positive regulation of myelination* pathway in old mice during SS (but not at ZT0). Also, downregulation of oligodendrocyte precursor cells (OPCs) and neuron marker genes were observed in the old mice during sleep.

Comparing sleep and wake (Figure 6b), upregulation of astrocyte genes was detected during SD in both ages. Upregulation of the oligodendrocyte genes during SD was observed in young mice only, while upregulation of the microglia genes during SS was observed in old mice only.

## 3 DISCUSSION

### 3.1 Summary

This is the first RNA‐Seq study comparing sleep/wake transcriptomic regulations between young and old mice in mPFC. When examining expression differences between SD and SS at four time points over the lights‐on period (ZT0–ZT12), we identified 5,158 DEGs in young mice and 3,540 DEGs in old mice at an FDR of 1%. Compared to our prior microarray study of cortex of young mice (Mackiewicz et al., 2007), this study not only identified a larger number of DEGs (5,158 vs. 3,988), but also detected larger changes (20% vs. 5% of DEGs with >50% change). This agrees with other studies comparing the two platforms (Ozsolak & Milos, 2011).

After restricting to genes with at least 15% change, we found 3,802 DEGs in young and 2,532 DEGs in old mice; roughly equal numbers of genes were upregulated by SD (wake genes) or SS (sleep genes). Thus, there is a 33% reduction in the number of sleep and wake DEGs in the old mice. This reduction in the number of DEGs is driven by a reduction in the magnitude of expression changes between sleep and wake in old mice, rather than increased variability with aging, including in ~70% of common DEGs differentially regulated in both ages.

While we found an overall reduction in expression changes between sleep and wake in old mice, not all pathways showed age‐related effects. Thus, it cannot simply be ascribed to alterations in the nature of sleep. In general, more wake‐activated functions were commonly enriched in young and old mice, such as *ER stress*, *regulation of transcription*, and *MAPK signaling*, than sleep‐activated functions, such as *DNA repair*. Also, many more sleep‐activated genes/functions, such as *synaptic transmission*, *synaptogenesis‐related cell adhesion*, *axon guidance, and neurogenesis*, were no longer differentially regulated in the old mice. Thus, transcriptional changes during sleep are particularly attenuated in old mice.

### 3.2 Homeostatic synaptic plasticity

Our data suggest synaptic homeostasis upscaling on the mRNA level is intact in old mice, as indicated by the SD upregulation of neuronal activity markers (e.g., *Fos*, *Arc*, and *Bdnf*) in both ages at nearly identical magnitudes. We also did not observe any expression difference of *Arc* and *Fos* between young and old mice during undisturbed sleep. Some studies observe age‐related reduction of *Arc* and *Fos* or response of *Fos* to stress in select brain regions or cell types (Kovacs, Schiessl, Nafz, Csernus, & Gaszner, 2018; Naidoo et al., 2011; Penner et al., 2011), while others show no differences (Desjardins et al., 1997), indicating the effect of age on neuronal activity markers is region or stimulus dependent. However, we did observe age‐related changes in sleep/wake regulations of some synaptic plasticity and synaptogenesis genes. Many of them have increased expression during sleep and decreased expression during SD in only the young mice, with no change in old mice. These genes include voltage‐gated calcium channel subunits (*Cacna1b* and *Cacna2d1*), known regulators of NMDARs and neurotransmitter release (Scheuber, Miles, & Poncer, 2004); piccolo (*Pclo*), synapsin (*Syn1* and *Syn2*), and neurexin (*Nrxn1* and *Nrxn3*), important players in presynaptic assembly (Chia, Li, & Shen, 2013); and reelin (*Reln*), an essential gene for neuronal development (Wasser & Herz, 2017). Age‐related reduction of *Reln* and its correlation with cognitive decline in rats has been shown (Stranahan, Haberman, & Gallagher, 2011).

Collectively, these results suggest two distinct impacts of aging on synaptic homeostasis. On the one hand, the capability of synaptic scaling‐up in response to extended wakefulness is intact in the mPFC of old mice. This agrees with a recent study showing local cortex neuron activation during SD was not affected by aging (McKillop et al., 2018). On the other hand, genes activated during sleep, particularly those involved in synaptic transmission and synaptogenesis, were often no longer regulated in old mice. This is consistent with studies where age‐associated impairments were evidenced in synaptic function and plasticity (Sama & Norris, 2013).

### 3.3 DNA repair

Sleep is important to repair DNA damage caused by increased neuronal activity during wake (Bellesi et al., 2016). We found that DNA repair pathways were among the most enriched for sleep genes in both young and old mice. However, the genes involved in these pathways generally showed lower increases during sleep compared to wake in old mice. Also, many wake‐active cell cycle genes that promote DNA repair (*Cdkn1a*, *Ccnd2*, and *Gadd45a*) (Hustedt & Durocher, 2016) were less expressed in old versus young mice during sleep or at baseline. Relatedly, a prior study has shown age‐dependent increase in DNA damage in humans >40 years old, particularly in genes central to synaptic function and plasticity (Lu et al., 2004). Our data suggest that age‐related increases in DNA damage might be in part due to insufficient sleep‐dependent DNA repair found in old mice.

### 3.4 Cholesterol synthesis

Prior microarray studies report that sleep is related to increased cholesterol synthesis in multiple brain regions (Mackiewicz et al., 2009). Cholesterol is mainly synthesized by oligodendrocytes and astrocytes in the brain, and the majority of cholesterol in the brain is used for myelin formation (Zhang & Liu, 2015). While the *cholesterol synthesis* pathway was not enriched among sleep genes in our study, several key enzymes involved in the synthesis of cholesterol from acetyl‐CoA/mevalonate (*Lss*, *Nsdhl*, *Fdft1*, *Fdps, Dhcr7*, and *Mvd*) were upregulated with sleep in young and old mice. Greater changes during sleep were generally observed in old mice. Moreover, we identified enrichment of *lipid metabolic process* and *positive regulation of myelination* among upregulated sleep genes in old mice. Oligodendrocytes marker genes were also significantly increased in old versus young mice during sleep. These results are consistent with prior studies. For example, studies have found age‐related increases in oligodendrogenesis and myelinogenesis in the spinal cord of mice (Lasiene, Matsui, Sawa, Wong, & Horner, 2009) and increases in the number of oligodendrocytes in prefrontal cortex of normal humans with aging were abolished in schizophrenia patients (Vostrikov & Uranova, 2011). Together, our results suggest an increased need for cholesterol to support myelination with aging, and that sleep disruption may contribute to myelin loss and, thus, neurological dysfunction.

### 3.5 Translation regulation

Sleep has been implicated in regulating protein synthesis. While no *translation regulation* pathway was enriched among our sleep genes, a few genes involved in protein synthesis were significantly upregulated by sleep. In particular, multiple eukaryotic translation initiation factors and mRNA processing genes (*Eif4b*, *Eif5*, *Rbm3*, and *Denr*) were induced by sleep in both ages, as seen previously in cortex of young mice (Mackiewicz et al., 2007). However, *Eif4E2*, a subunit of the rate‐limiting translation factor *Eif4E* (Rau, Ohlmann, Morley, & Pain, 1996), was upregulated by SD in our study. Eukaryotic elongation factor 2 (*eEF2*), an essential factor for protein synthesis (Taha, Gildish, Gal‐Ben‐Ari, & Rosenblum, 2013), was not regulated by sleep in mPFC of mice. This suggests a possible region‐specific effect of sleep/wake on translation regulation.

### 3.6 ER stress

Sleep deprivation‐induced ER stress is shown in different brain regions and across multiple species (Brown & Naidoo, 2010). Here, we found *ER stress/unfolded protein response* among the most enriched pathways for genes upregulated by SD. Many of the key ER stress genes from microarray studies (e.g., *Hspa5* and *Dnajb5*) (Mackiewicz et al., 2009) were upregulated with SD in both young and old mice. Previous studies show that SD induction of *Hspa5* (*BiP*) protein is not present in aged mice (Naidoo, 2009). However, our study shows *Hspa5* is similarly induced by SD in old and young mice on the transcript level. A similar result has been shown using real‐time PCR in mouse hippocampus (Vecsey, Park, Khatib, & Abel, 2015). On the other hand, we observed a statistically significant 6% reduction in SD‐related FCs among all genes (*n* = 57) involved in ER stress in old mice, suggesting a subtle attenuation at the pathway level with aging.

### 3.7 Age‐related upregulation of immune system and microglia genes

Consistent with prior gene expression studies with aging (Mohan, Mather, Thalamuthu, Baune, & Sachdev, 2016), we found enrichment of *inflammatory/immune response* pathways in gene upregulated in old mice. Age‐related increases in the number of microglia have been found in multiple brain regions in mice (Wong, 2013). Here, marker genes for microglia were also increased in old mice. Several of the most highly induced genes from the microglia gene sets (*Clec7a*, *Cybb*, *Itgax*, and *Csf1*) are specific markers of the “primed” microglia, a status associated with aging and neurodegenerative diseases (Holtman et al., 2015). Primed microglia is also linked to upregulation of lysosome and antigen presentation (Holtman et al., 2015). Consistent with this, our data also found an upregulation of multiple lysosome genes (*Lyz2* and *Litaf*) and MHC class II genes (*H2‐Q6* and *H2‐Q7*) in old mice. As several studies link microglia activation with neurogenesis inhibition and synapse pruning (Reshef et al., 2017), increased activation of microglia genes in old mice particularly during sleep may explain a reduction in neurogenesis and synaptogenesis with aging.

### 3.8 Age differences in sleep/wake regulation of oligodendrocyte and astrocyte genes

Besides microglia, we observed a positive enrichment of oligodendrocyte genes in old mice. Studies have shown an important cross talk between microglia and oligodendrocytes (Peferoen, Kipp, van der Valk, van Noort, & Amor, 2014). Other than immune functions, oligodendrocytes play important roles in stress response and may aid in neuronal protection and regeneration. A study of SD effects on isolated oligodendrocytes showed upregulation of apoptosis and cellular stress response genes in oligodendrocytes (Bellesi et al., 2013). Interestingly, our data revealed an enrichment of SD‐induced (or sleep‐depressed) oligodendrocyte marker genes in young mice only. Several well‐studied oligodendrocyte marker genes (e.g., *Cryab*, *Hapln2*, *Mbp*, *Mobp*, *Anln*, and *Plxnb3*) were downregulated during sleep only in young mice; thus, expression of these genes was elevated in old mice during sleep compared to young mice, suggesting potential roles of sleep/wake regulation in oligodendrocyte‐related neuronal protection.

Astrocytes are the main supportive cells of the brain, and play a key role in the homeostatic control of sleep and wake (Fellin, Ellenbogen, De Pitta, Ben‐Jacob, & Halassa, 2012). We saw upregulation of astrocyte marker genes with SD in both ages. This is consistent with results for synaptic homeostasis genes (*Arc* and *Bdnf*), again suggesting the molecular homeostatic response to forced wakefulness is not affected by aging. Results are supported by a recent study revealing unchanged expression of isolated astrocyte homeostasis genes with aging (Boisvert, Erikson, Shokhirev, & Allen, 2018). One exception is *Gfap*, an astrocyte marker gene that was downregulated during SS in young mice only and showed elevated expression in old mice during sleep. *Gfap* is a marker of reactive astrogliosis, which is associated with age‐related neuroinflammation (Boisvert et al., 2018). Thus, our data suggest that sleep lessens neuroinflammation related to activated astrocytes and that aging attenuates this effect.

### 3.9 Limitations

A limitation of our study is that it does not evaluate gene expression during the dark phase. Thus, data are inadequate to evaluate the impact of age in the circadian oscillation of gene expression. However, when examining the 12 core clock genes, we found all but two (Per3 and Nr1d1) were significantly different between SS and SD in young mice. In the old mice, only five of these genes were significantly regulated, suggesting a possible age‐related change on the effect of sleep/wake on circadian gene expression. A second limitation is SD via gentle handling can potentially introduce mild stress in addition to wakefulness. However, earlier study on rat (Cirelli, 2002) found similar pathways/genes being differentially regulated when comparing sleep to either SD or spontaneous wakefulness in young animals. Additionally, key pathways we described as different between sleep and SD are also found in a previous study where mice had cortisol levels maintained at a constant level after adrenalectomy (Mongrain et al., 2010). While spontaneous wakefulness can avoid possible stress introduced by gentle handling, mice will need to be harvested in the early dark phase, where sleeping controls at matching time point are very difficult to obtain. Additionally, aged mice are known to have decreased wakefulness during the dark phase (Wimmer et al., 2013); therefore, assessment of the effect of age on wakefulness at the dark phase is likely to be confounded by the mismatch in the amount of wakefulness between age‐groups. The current study design, which we have used previously (Anafi et al., 2013; Mackiewicz et al., 2007), allows the two age‐groups to be compared with similar amounts of sleep (using infrared beam monitoring) and wake (total SD) at matching diurnal time points. Therefore, we chose to use SD instead of spontaneous wakefulness. Future studies are of interest to examine the potential impact of mild stress on the effect of age on sleep/wake regulation. Finally, we noted that our study replicate results from earlier transcriptomic studies either on the effect of sleep/wake (in young animals) or on aging. As this is the first RNA‐Seq study describing the effect of age on sleep/wake controls, future independent studies replicating the novel findings of our study, ideally using RNA‐Seq, will be beneficial to the field.

### 3.10 Conclusions

Using RNA sequencing in mPFC, we identified a large number of differentially regulated genes impacted by sleep and wake in young and old mice. There was a system‐wide reduction in the number and magnitude of expression changes of the transcript level in old mice, suggesting an age‐associated attenuation in their sleep/wake regulation. Specifically, our study revealed that sleep‐activated processes (e.g., DNA repair, synaptogenesis, axon guidance, and neurogenesis) were sensitive to the effect of aging. The magnitude of change was attenuated in old mice. These results provide potential molecular targets for studying the connections between age‐related sleep disorders and neurodegenerative diseases.

## 4 EXPERIMENTAL PROCEDURES

Additional details and references are presented in the Online Supplement.

### 4.1 Mouse experiments

Experiments were performed on male C57BL/6 mice at 2–4 months (young) and 18–20 months (old) housed individually in a 12/12 light/dark room. Sleep deprivation was initiated with gentle handling at lights‐on (7 a.m.; ZT0) and continued for 3, 6, 9, and 12 hr in different groups. Time‐matched SS mice with ≥75% sleep in both ages in the last hour before sacrifice were collected. Randomly selected mice were sacrificed at ZT0 for baseline data. Six mice were collected in each condition.

### 4.2 RNA purification, sequencing and bioinformatics

RNA extraction was performed from microdissected mPFC using an RNAqueous‐Micro Kit. Library preparation was performed using Illumina TruSeq Stranded mRNA kit and sequencing done with 100‐base pair single‐end reads on Illumina HiSeq 4,000. Raw reads were aligned to the mouse genome build mm9 by STAR version 2.5.3a, and quantified at the gene level using scripts from the PORT pipeline (github.com/itmat/Normalization‐v0.8.4‐beta). A total of 14,656 genes with unique ensemble IDs passed filtering [mean log_2_ counts per million (CPM) > −1.4] and were normalized using the “Trimmed Mean of M‐values” (TMM) method.

### 4.3 Differential Expression Analysis and Functional Analysis

We evaluated DEGs between SD and SS within each age‐group and between ages (in undisturbed conditions, i.e., SS and ZT0) using LimmaVoom. For each gene, a moderated *F* test was used to evaluate an ANOVA‐like null hypothesis of any pairwise differences between conditions (or ages) within each of the four separate time points. False discovery rate was controlled at 1% using the Benjamini–Hochberg method. To test if age modified the differences between SS and SD at any of the time points, we used statistical interaction tests; given lower power for interactions, an FDR threshold of 5% was used. Unless otherwise noted, FDR values and FCs between young and old mice were compared using Wilcoxon signed‐rank tests. DAVID was used for gene ontology (GO) enrichment analyses and functional clustering. Gene set enrichment tests were performed using CAMERA with Limma. Cell‐type‐specific marker gene sets (Table S9) were obtained from microarray study of isolated cell types and single‐cell RNA‐Seq studies.

## CONFLICT OF INTEREST

None declared.

## AUTHORS' CONTRIBUTION

A.I.P. designed the study and was involved in writing and editing. X.G. contributed to data collection, analysis, writing, and editing. B.T.K. contributed to data analysis, writing, and editing. D.S. and G.R.G. contributed to data analysis. D.C.L. contributed to data collection and editing. J.L. contributed to data collection.

## Data Availability

All raw and processed RNA‐Seq data are available at Gene Expression Omnibus (GEO) (GSE128770).
