# Supplementary material for: Age attenuates the transcriptional changes that occur with sleep in the medial prefrontal cortex
Source: Aging Cell. 2019 Sep 24;18(6):e13021. doi: 10.1111/acel.13021 (PMC6826131; doi:10.1111/acel.13021)
Supplement: Supplementary file 1 [file ACEL-18-e13021-s001.pdf]

Supplemental material

EXPERIMENTAL PROCEDURES (full version)

4.1 Mouse experiment

Experiments were performed on male C57BL/6 mice in two age groups – young mice at 2-4 months of age and old mice at 18-20 months of age. Animals were housed individually on a 12hour/12hour light/dark cycle with lights-on at 7AM (Zeitgeber time zero or ZT0), in a pathogen-free, temperature (22°C), and humidity (45-55%) controlled room. Water was available *ad libitum*. Food was available only during the 12 hours of the lights off period (active period), as described previously [1, 2], to avoid potential food intake differences between the mice that were sleep deprived and the mice that were allowed to sleep during the last lights on period of the experiment [3, 4]. Animals were acclimated for 14 days to the food restriction regime and the presence of experimenter prior to the experiment. All mice (n=54 for each age group) were behaviorally monitored with the AccuScan infrared monitoring system that detects movement when the mouse crosses electronic beams. To assess behavior prior to sacrifice, based on previous validation studies with EEG/EMG, sleep was defined as ≥40 seconds of continuous inactivity in young mice [5] and as ≥50 seconds of continuous inactivity in old mice [6]. On the days of the experiments, sleep deprivation (SD) was initiated using gentle handling [7] at lights-on, and young and old animals were collected following 3, 6, 9, and 12 hours of SD (n=6 at each time point)(3). For mice that had been allowed spontaneous sleep (SS), their sleep was monitored starting from lights-on, and only mice that slept for ≥75% of the last one hour and ≥60% of the last three hours before sacrifice were included in the SS group. Additional groups of young and old mice (n=6 for each age) were also chosen randomly at ZT0 to use for “baseline” assessment of gene expression. All experiments were approved by the Institution of Animal Care and Use

Committee of the University of Pennsylvania and were carried out in accordance with all National Institutes of Health guidelines.

4.2 Tissue collection and RNA purification

Mice were euthanized by cervical dislocation and brains were quickly removed, rapidly frozen on dry ice, and stored at -80°C. Microdissection of the frozen brain was performed in a cryostat using micro-punch needles following a process previously described [8, 9]. Briefly, medial prefrontal cortex (mPFC), including anterior cingulate, pre-limbic, and infra-limbic prefrontal cortex, was dissected from five 300µm consecutive sections between approximately 2.0 to 0.5mm Bregma according to the mouse brain atlas from Allen Institute for Brain Science [10, 11]. The entire micro-punching process was performed within the cryostat at -20°C. Once cut, brain sections were laid flat sequentially on a clean glass slide that had been pre-cooled in the cryostat, and one punch on each brain slide using a 1.5mm Harris Uni-Core micro-sampling tool (GE Healthcare) was made and the dissected mPFC regions were ejected into a RNase-free microcentrifuge tube (Invitrogen) that had been pre-cooled on dry ice. The dissected tissues were stored at -80°C until RNA extraction. RNA extraction was performed using an RNAqueous-Micro Total RNA Isolation Kit (Invitrogen) and on-column DNase digestion was performed using an RNase-Free DNase Set (Qiagen) following the manufacturer's protocol. RNA concentration and purity were measured by Nanodrop 1000 spectrophotometer. RNA integrity (RIN) was measured with Agilent 2100 Bioanalyzer RNA 6000 Nano chip. All samples had RIN values above 8.0, with a mean (standard deviation) RIN of 9.05 (0.36).

4.3 Sequencing and bioinformatics

RNA-Seq library preparation was performed by the University of Pennsylvania Next-Generation Sequencing Core using a TruSeq Stranded mRNA kit (Illumina) and sequencing was done with 100 base-pair single-end reads on a HiSeq 4000 System (Illumina). On average (standard deviation), 24.5 (2.6) million total raw reads were obtained for each sample. Raw RNA-Seq reads were aligned to the mouse genome build mm9 by STAR version 2.5.3a [12]. After the alignment, an average of 17.3 (1.9) million reads were mapped uniquely to annotated genes. The aligned data were quantified at the gene level using scripts from the PORT pipeline (github.com/itmat/Normalization -v0.8.4-beta). Fragments from the same strand as the annotated genes were identified as sense, and fragments from the opposite strand of the annotated genes were identified as antisense. Low-expressing genes were removed by keeping only the genes with mean $\log_2$ counts per million (CPM) >-1.4 (equivalent to 5 counts with a library size of 15 million reads). A total of 14,656 genes with unique ensemble IDs that passed the filtering criteria were normalized using the "Trimmed Mean of M-values" (TMM) method in the edgeR package [13], and differential expression analyses were performed using the LimmaVoom package [14] (see also below). A multidimensional scaling (MDS) plot of all samples was generated using the plotMDS function in Limma with default parameters. The Euclidean distance between each pair of samples, calculated as the root-mean-square deviation of the top 500 most variable genes automatically selected for each pair of samples, was used as an indication of the similarity between samples [14]. The first dimension represents the leading-fold-change that best separates samples and explains the largest proportion of variation in the data, with subsequent dimensions having a smaller effect and being orthogonal to the ones before it.[15]

4.4 Assessment of Differential Expression

Gene expressions were measured at baseline (ZT0, 7AM, lights-on) in each age group (young, old) and at 4 time-points [ZT3 (10AM), ZT6 (1PM), ZT9 (4PM), ZT12 (7PM)] in each age-group and behavioral state (SS, SD). Thus, there were a total of 18 experimental conditions defined by age and behavioral state. Using the LimmaVoom package in R software [14], we evaluated the differentially expressed genes (DEGs) between sleep deprived and spontaneous sleep behavioral states (SD vs. SS) within each age group. Differentially expressed genes between young and old (old vs. young) were only evaluated in the undisturbed conditions (SS and ZT0). For each of the 14,656 genes that passed through the filtering criteria, statistical significance evaluating differentially expressed genes between behavioral state or age-group was based on a moderated F-test [14, 16] testing for any significant differences at any of the four time-points (e.g., an ANOVA-like test). Similarly, comparisons between young and old mice at baseline (ZT0) were performed using a moderated T-test (given a single time-point for comparison). Multiple comparisons correction for statistical significance was applied using the method of Benjamini and Hochberg [17] to control the overall false discovery rate (FDR) at 1%, unless otherwise noted.

Statistical interaction was tested between behavioral state and age group for the identified sleep genes and wake genes from both young and old animals combined. Analyses were performed in a similar manner to those described above, using a moderated F-test to assess whether the differences in gene expression between SS and SD differed between young and old mice at any of the four timepoints. Multiple comparison correction using Benjamini and Hochberg was used

and genes with FDR less than 5% were considered significant for the interaction test; a more liberal FDR threshold was chosen given the expected reduced power to detect interaction effects.

Unless otherwise noted, the statistical tests comparing FDR values and fold changes of genes between young and old animals were performed using paired Wilcoxon signed-rank tests. Effect sizes were calculated using Cohan's d method [18].

4.5 Functional analysis

DAVID Bioinformatics Resources 6.8 was used for gene ontology (GO) enrichment analyses [19, 20]. Unless otherwise noted, enrichment of functional annotation was performed against four databases: *GOTERM_BP_Direct* (biological process), *GOTERM_MF_Direct* (molecular function), UP_keywords (UniProt keyword), and KEGG pathways. The 14,656 genes used for generating DEGs were used as the background in all cases. Enrichment of individual terms was defined as a $P<0.05$ and containing at least 3 genes. Functional clustering of overlapping terms was performed with kappa similarity threshold >0.3, minimum similarity terms≥3, multiple linkage threshold >0.3, and EASE <0.05. The significance of the enriched functional clusters was evaluated using an enrichment score, which is equivalent to the $-\log_{10}$ transformed geometric mean of the Ps of included functional annotation terms. Since only terms with $P<0.05$ are included in the clusters, the minimum enrichment score from enriched functional clusters is 1.3.

4.6 Gene set enrichment analysis

Competitive gene set enrichment tests were performed using the CAMERA [21] method in the Limma package [14]. The default setting of inter.gene.corr=0.01 was used for all tests. Enrichment of the cell-type specific genes was done by matching customized gene sets containing lists of cell-type specific marker genes against the ranked fold-changes of different comparisons (e.g., SD vs. SS and Old vs. Young), as described above. Cell-type specific marker gene sets were obtained from three studies, a microarray study performed using isolated astrocytes, neurons, and oligodendrocytes from mouse forebrain by Cahoy et al. [22], a single-cell RNA-seq study performed using cells dissociated from mouse primary visual cortex by Tasic et al. [23], and a single-cell RNA-seq study performed with the use of a cocktail of inhibitors blocking neuronal activation caused by enzyme digestion during the dissociation process by Hrvatin et al. [24]. **Table S9** lists the sources and selection criteria of the cell-type specific gene sets, as well as the full lists of the genes included in the gene sets.

1. Mackiewicz, M., et al., *Macromolecule biosynthesis: a key function of sleep.* Physiol Genomics, 2007. **31**(3): p. 441-57.
2. Anafi, R.C., et al., *Sleep is not just for the brain: transcriptional responses to sleep in peripheral tissues.* BMC Genomics, 2013. **14**: p. 362.
3. Hatori, M., et al., *Time-Restricted Feeding without Reducing Caloric Intake Prevents Metabolic Diseases in Mice Fed a High-Fat Diet.* Cell Metabolism, 2012. **15**(6): p. 848-860.
4. Mavanji, V., et al., *Partial sleep deprivation by environmental noise increases food intake and body weight in obesity-resistant rats.* Obesity (Silver Spring), 2013. **21**(7): p. 1396-405.
5. Pack, A.I., et al., *Novel method for high-throughput phenotyping of sleep in mice.* Physiol Genomics, 2007. **28**(2): p. 232-8.
6. Naidoo, N., et al., *Aging impairs the unfolded protein response to sleep deprivation and leads to proapoptotic signaling.* Journal of Neuroscience, 2008. **28**(26): p. 6539-6548.
7. Franken, P., et al., *Sleep-Deprivation in Rats - Effects on Eeg Power Spectra, Vigilance States, and Cortical Temperature.* American Journal of Physiology, 1991. **261**(1): p. R198-R208.
8. J, P.M.a.B.M., *Microdissection of Brain Areas by the Punch Technique*, in *Microdissection of Brain Areas by the Punch Technique*, C.A. C, Editor 1983, Wiley: Chichester. p. 1-36.
9. Palkovits, M., *Microdissection of Individual Brain Nuclei and Areas*, in *General Neurochemical Techniques*, A.A.B.B. Baker, Editor 1985, Humana Press: Totowa, NJ.
10. Bakker, R., P. Tiesinga, and R. Kotter, *The Scalable Brain Atlas: Instant Web-Based Access to Public Brain Atlases and Related Content.* Neuroinformatics, 2015. **13**(3): p. 353-366.

11. Lein, E.S., et al., *Genome-wide atlas of gene expression in the adult mouse brain.* Nature, 2007. **445**(7124): p. 168-176.
12. Dobin, A., et al., *STAR: ultrafast universal RNA-seq aligner.* Bioinformatics, 2013. **29**(1): p. 15-21.
13. Robinson, M.D., D.J. McCarthy, and G.K. Smyth, *edgeR: a Bioconductor package for differential expression analysis of digital gene expression data.* Bioinformatics, 2010. **26**(1): p. 139-140.
14. Ritchie, M.E., et al., *limma powers differential expression analyses for RNA-sequencing and microarray studies.* Nucleic Acids Research, 2015. **43**(7).
15. Law, C.W., et al., *RNA-seq analysis is easy as 1-2-3 with limma, Glimma and edgeR.* F1000Res, 2016. **5**.
16. Peart, M.J., et al., *Identification and functional significance of genes regulated by structurally different histone deacetylase inhibitors.* Proceedings of the National Academy of Sciences of the United States of America, 2005. **102**(10): p. 3697-3702.
17. Benjamini, Y. and Y. Hochberg, *Controlling the False Discovery Rate - a Practical and Powerful Approach to Multiple Testing.* Journal of the Royal Statistical Society Series B-Methodological, 1995. **57**(1): p. 289-300.
18. Cohen, J., *Statistical Power Analysis for the Behavioral Sciences*1988, New York: NY: Routledge Academic.
19. Huang, D.W., B.T. Sherman, and R.A. Lempicki, *Systematic and integrative analysis of large gene lists using DAVID bioinformatics resources.* Nature Protocols, 2009. **4**(1): p. 44-57.
20. Huang, D.W., B.T. Sherman, and R.A. Lempicki, *Bioinformatics enrichment tools: paths toward the comprehensive functional analysis of large gene lists.* Nucleic Acids Research, 2009. **37**(1): p. 1-13.
21. Wu, D. and G.K. Smyth, *Camera: a competitive gene set test accounting for inter-gene correlation.* Nucleic Acids Research, 2012. **40**(17).
22. Cahoy, J.D., et al., *A transcriptome database for astrocytes, neurons, and oligodendrocytes: A new resource for understanding brain development and function.* Journal of Neuroscience, 2008. **28**(1): p. 264-278.
23. Tasic, B., et al., *Adult mouse cortical cell taxonomy revealed by single cell transcriptomics.* Nature Neuroscience, 2016. **19**(2): p. 335-+.
24. Hrvatin, S., et al., *Single-cell analysis of experience-dependent transcriptomic states in the mouse visual cortex (vol 21, pg 120, 2018).* Nature Neuroscience, 2018. **21**(7): p. 1017-1017.

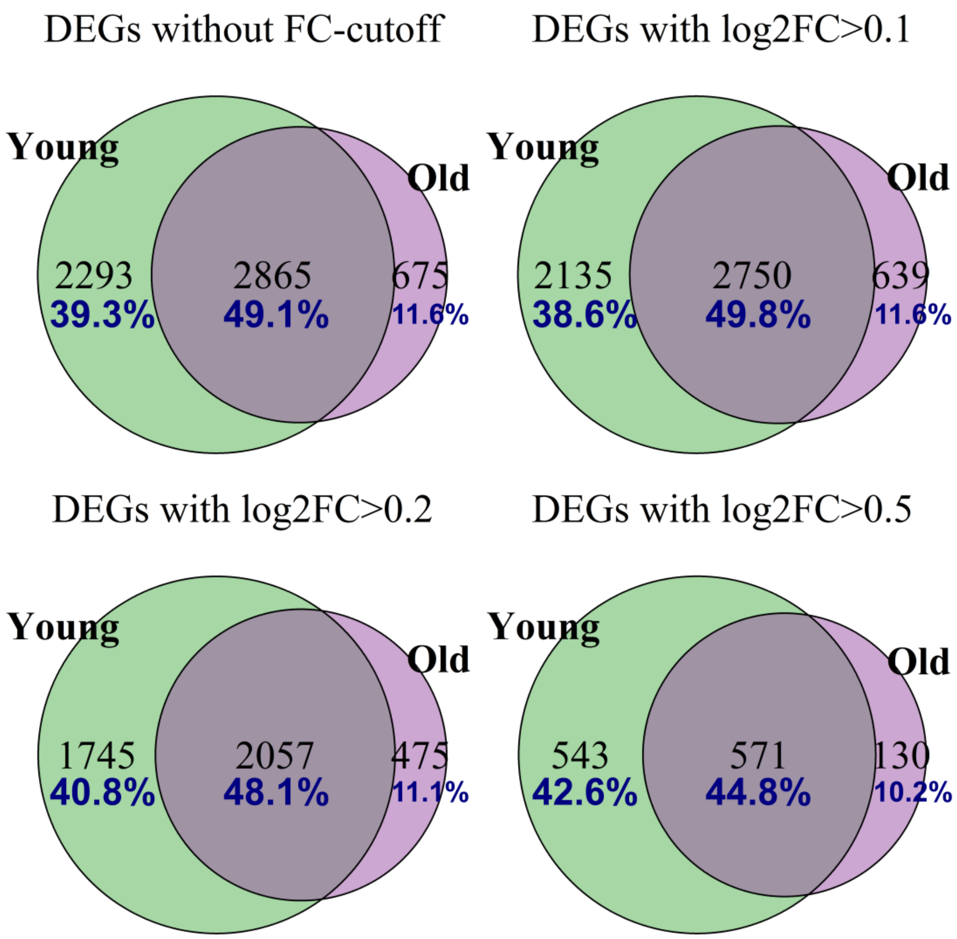

Figure S1. Venn diagram of the DEGs identified between SD vs. SS within the young and the old mice across timepoints (FDR<1%) after applying varying fold-change (FC) cutoffs (based on absolute log2FC averaged across four time-points). Numbers of DEGs passing FC-cutoffs decrease as the FC-cutoff threshold increases from zero (without FC-cutoff) to 0.1, 0.2, and 0.5 for both young and old mice. But the proportions of genes being common or age-specific between the young and old mice stay relatively unchanged.

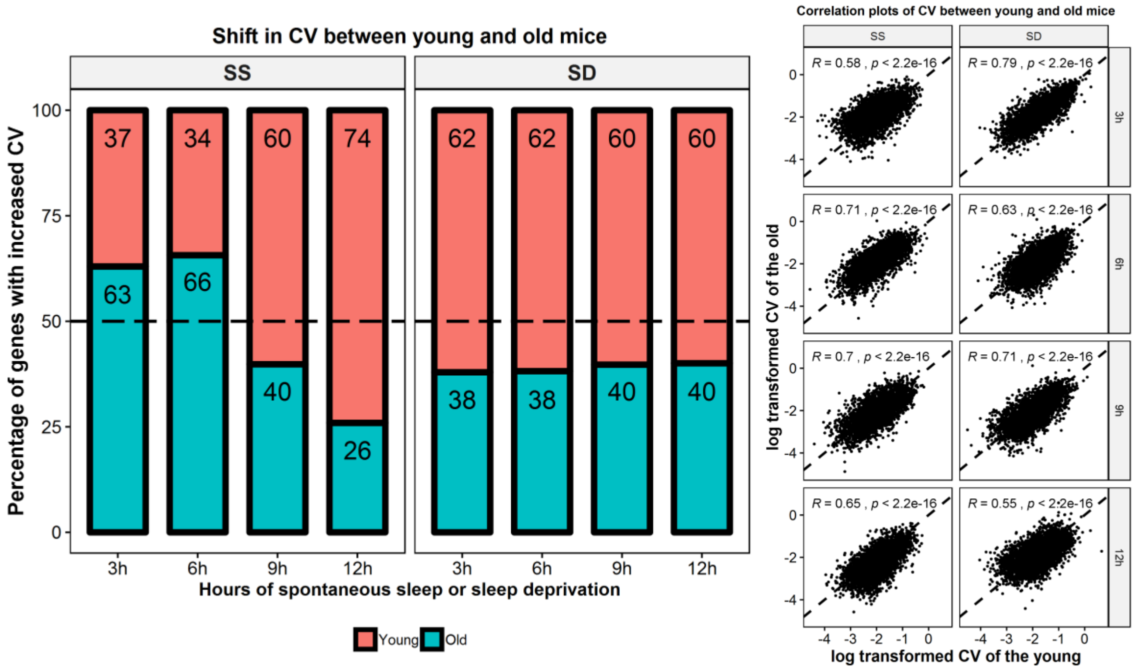

Figure S2. Variability of the gene expressions (counts per million) for the sleep and wake genes among biological replicates (n=6) was measured using coefficient of variance (CV) during sleep (left) and SD (right) and compared between young and old animals. (A) CVs are highly correlated between young and old animals at all the conditions tested. (B) stacked bar plots of the percentage of genes have increased CV in young (top) or old (bottom) mice. At only two of the eight condition (3h and 6h of SS) more than half of the genes (around 65%) showed increased CV in the old animals compared to the young animals. In the remaining six conditions (9h and 12h of SS, and SD at all four time points), 60% to 75% of the genes showed increased CV in the young animals than in the old animals.

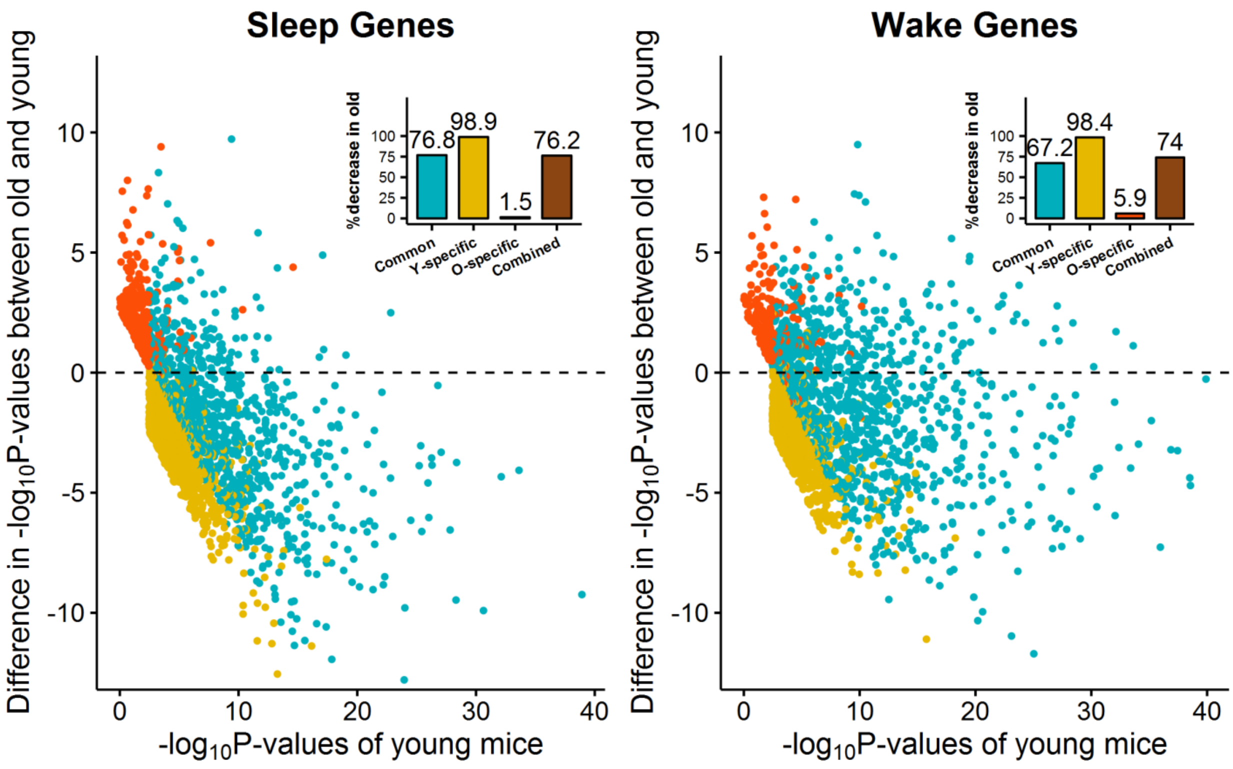

Figure S3. Scatter plots of the differences in $-\log_{10}$ P-values of individual sleep (left) and wake (right) genes between old and young mice. Positive differences indicate increased significance in old mice, and negative differences indicate decreased significance in old mice. The insets show the bar plots of percentage of genes in each gene class, common or age-specific, that have decreased significance in old mice (negative differences) compared to the young mice. Specifically, more than 98% of the young-specific sleep and wake genes, as well as 76.8% of common sleep genes and 67.2% of common wake genes are less significant in old mice, whereas 98.5% of the old-specific sleep genes and 94% of old-specific wake genes are more significant in old mice. The small percentages of age-specific genes showing increased significance in the opposite age group are likely due to the applied fold-change cutoff that allocates similarly significant genes into age-specific groups. Combined togther, 76.2% of sleep genes (1624 of 2130) and 74% of wake genes (1589 of 2147) are less significantly regulated between sleep and wake in old animals compared to the young animals.

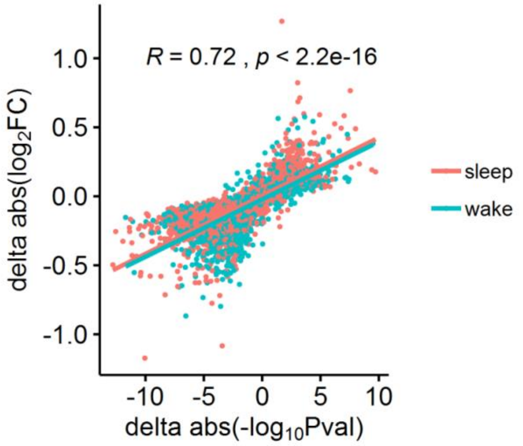

Figure S4. Correlation between the delta $-\log_{10}$ P-values and delta $\log_2$FC of individual sleep (red) and wake (blue) genes between old and young mice. Positive differences indicate increased significance or FC in old mice, and negative differences indicate decreased significance or FC in old mice. Combined pearson correlation coefficientce and p-value are shown.

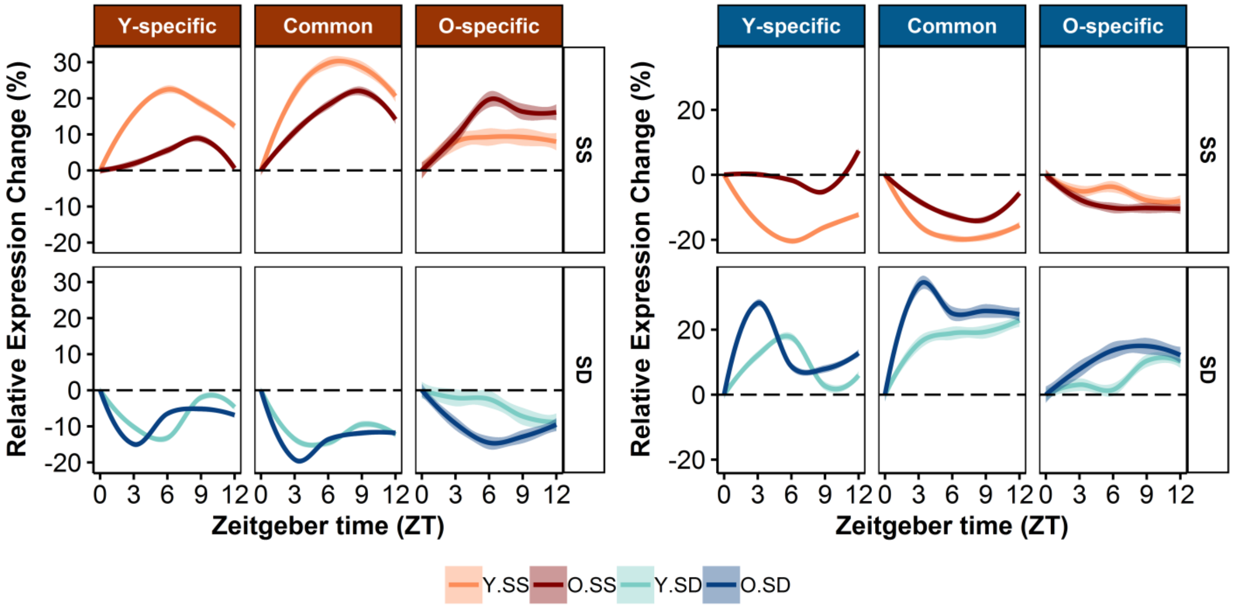

Figure S5. Percentages of expression changes relative to baseline (ZT0) of each gene during SS or SD were calculated and trend lines were plotted using Loess local regression (with 95% CI). Sleep genes were shown on the left (red headings) and wake genes were shown on the right (blue headings). Both common and young-specific genes showed a reduction in the magnitude of change between SS and ZT0 in the old mice compared to the young. Y.SS=young mice during SS; Y.SD=young mice during SD; O.SS=old mice during SS; O.SD=old mice during SD.

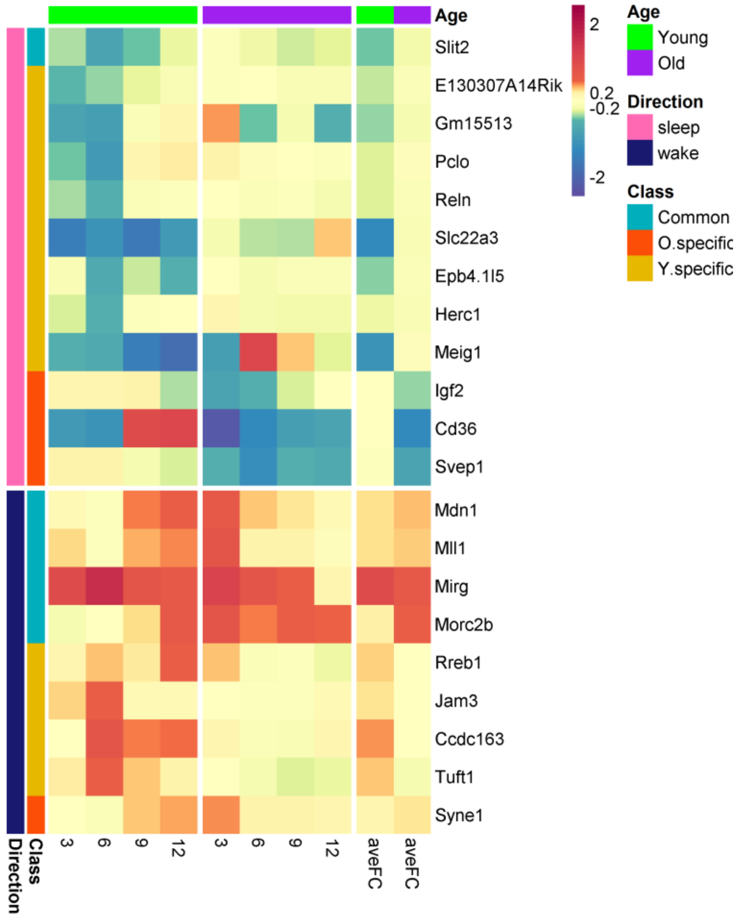

Figure S6. Heatmap of the log2 fold change between SD and SS for the 21 genes that are significant (FDR<5%) in the interaction tests (age*behavior state). Fold changes at each time point are shown for young (left) and old (center) mice, as well as the averaged fold changes (aveFC) for young and old mice across the four time points (right).The 12 sleep genes (top) and 9 wake genes (bottom) are further categorized into three classes, Y.specific, O.specific, or Common, based on if or not the genes are identified as sleep or wake genes in young animals, old animals, or both ages.

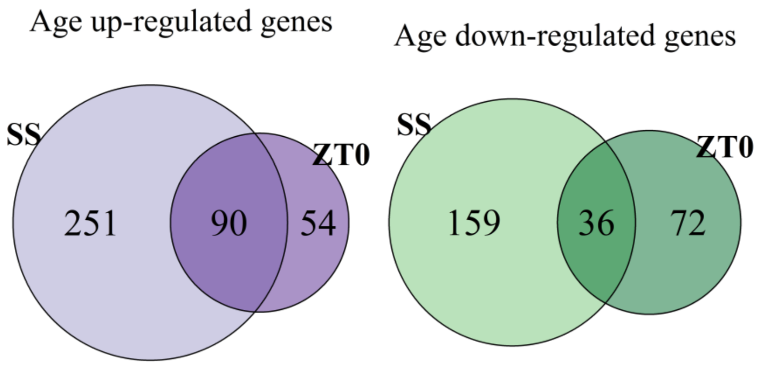

Figure S7. Venn diagram of the age up-regulated genes (left) and age down-regulated genes identified between young and the old mice during sleep (over the four time points) in spontaneous sleep (SS) group or at ZT0 (FDR<1%).

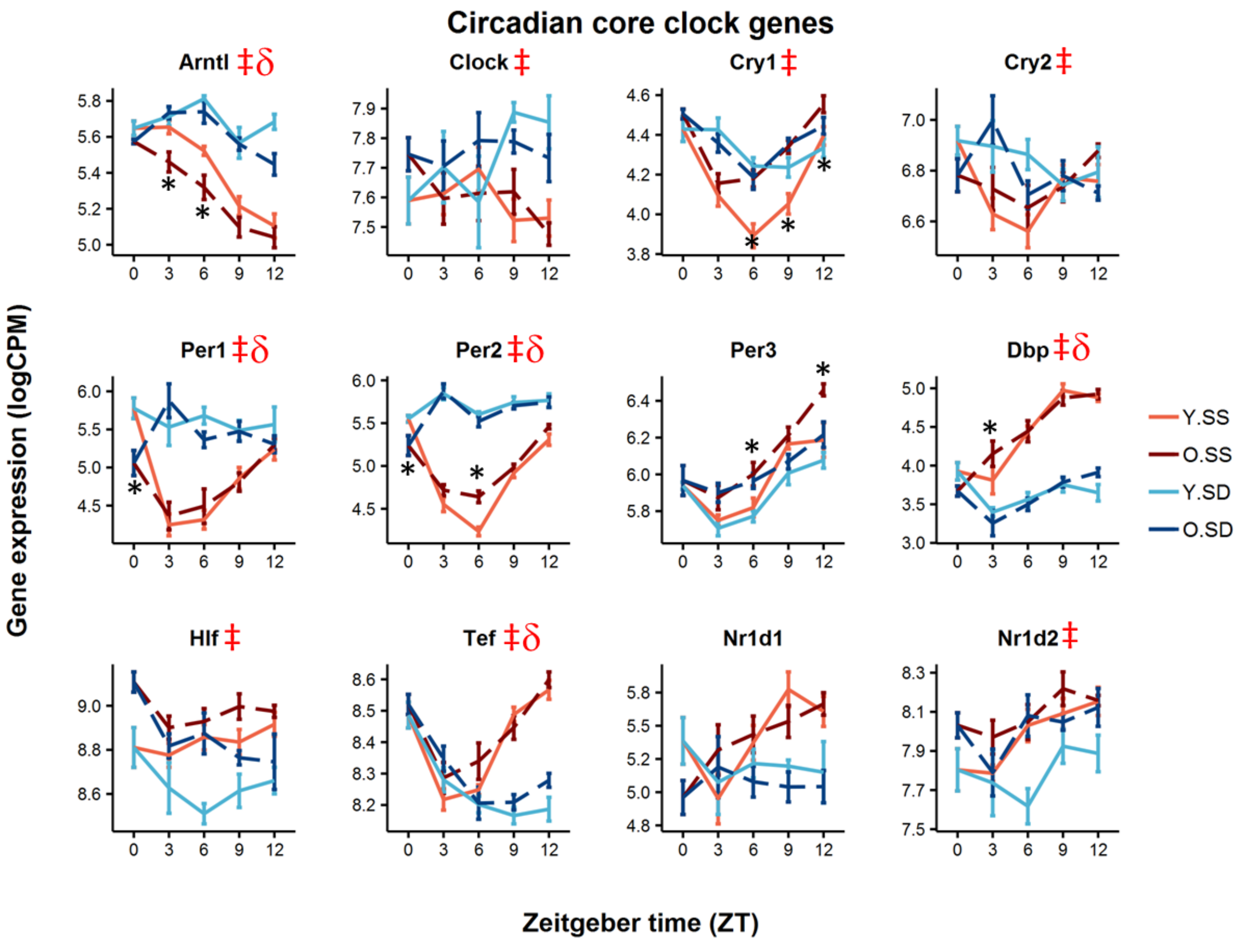

Figure S8. Expression profiles of 12 core clock genes during spontaneous sleep (SS) and SD in the young (solid line) and old animals (dotted lines). Five genes significantly (FDR<0.01) altered in both young and old mice (*Arntl*, *Per1*, *Per2*, *Dbp*, and *Tef*) are marked by ‡δ. Five additional genes only significantly (FDR<0.01) altered in young animals (*Clock*, *Cry1*, *Cry2*, *Hlf*, and *Nr1d2*) are marked by ‡. By comparing young and old mice during SS or at ZT0, five genes showed overall significant age differences using moderated F-test (Cry1: FDR<0.01; *Arntl*, *Per1*, *Per2*, and *Per3*: FDR between 0.01-0.05), and asterisk (*) at individual time point indicate a nominally significant difference (P.value<0.05) between young and old. The multiple comparison corrected FDR values were provided in **Table S3** for the moderated F-test of the SD vs SS contrasts over the four time-points for young and old mice, as well as the moderated F-tests of the O vs Y contrasts over the four time-points during SS and at ZT0.

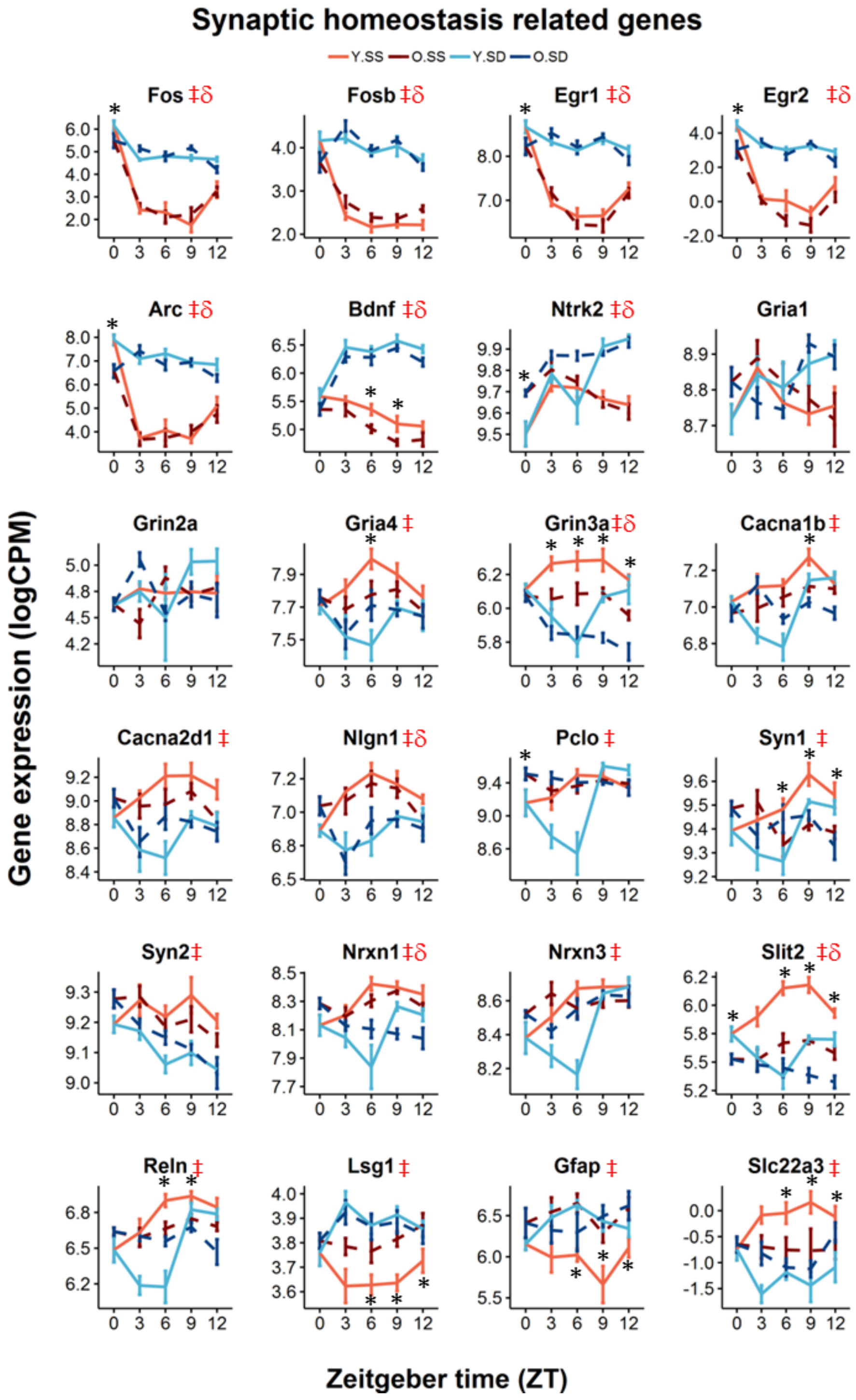

Figure S9. Expression profiles of genes related to synaptic homeostasis control during spontaneous sleep (SS) and SD in the young (solid lines) and in old animals (dotted lines). ‡ marks genes significantly altered between SD and SS in the young mice, and δ marks genes significantly altered between SD and SS in the old mice. Although many transcription factors that are indicators of neuronal activities (Fos, Fosb, Egr1, Egr2, Arc, Bdnf, Ntrk2) are similarly regulated between sleep and wake in both young and old animals, many other genes playing key roles in synaptic assembly and neurotransmitter release were

only regulated in the young but not in the old. Asterisk (*) at individual time point indicate a nominally significant difference (P.value<0.05) between young and old during SS or at ZT0. The multiple comparison corrected FDR values were provided in **Supplement Table 4** for the moderated F-test of the SD vs SS contrasts over the four time-points for young and old mice, as well as the moderated F-tests of the O vs Y contrasts over the four time-points during SS and at ZT0.

Table S4. Comparison of the mean fold changes between young and old mice of the enriched functions among sleep genes

source	Functions	Fold changes				Effect Sizes between young and old (d)		P-values	
		SD vs. SS		SS vs. ZT0					
		Young	Old	Young	Old	SD vs. SS	SS vs. ZT0	SD vs. SS	SS vs. ZT0
common	DNA repair	0.68±0.02	0.73±0.01	1.28±0.03	1.15±0.02	0.42	**0.71**	***1.4E-06***	***1.8E-04***
	Signal/transmembrane	0.73±0.01	0.76±0.01	1.21±0.01	1.12±0.01	0.27	0.45	***1.2E-19***	***4.0E-16***
	Gprotein receptor	0.71±0.02	0.77±0.02	1.19±0.02	1.07±0.03	0.48	**0.89**	***4.0E-06***	***5.0E-04***
Y-specific	Cell adhesion	0.8±0.01	0.9±0.01	1.16±0.01	1.01±0.02	**1.62**	**1.34**	***5.8E-14***	***4.5E-12***
	Axon guidance	0.81±0.01	0.89±0.01	1.15±0.01	0.99±0.01	**1.54**	**1.92**	***2.3E-09***	***8.8E-09***
	Signal/Transmembrane	0.79±0.005	0.88±0.004	1.15±0.01	1.02±0.01	**1.12**	**1.12**	***1.6E-48***	***3.0E-35***
	Kinase activity	0.8±0.04	0.93±0.03	1.07±0.05	0.94±0.03	**1.67**	**1.79**	0.125	0.125
	Chloride channel	0.8±0.03	0.89±0.01	1.16±0.04	0.99±0.01	**1.03**	**2.04**	***0.004***	***0.008***
O-specific	Collagen/glycoprotein	0.88±0.01	0.76±0.01	1.1±0.03	1.12±0.02	**1.05**	0.15	***1.5E-15***	0.094
	Transmembrane	0.88±0.01	0.76±0.01	1.11±0.02	1.13±0.02	**1.07**	0.15	***1.6E-20***	***0.040***
	Calcium transport	0.87±0.02	0.75±0.02	1.07±0.03	1.13±0.03	**1.04**	0.35	***1.9E-09***	0.077
*	Transmem.combined	0.78±0.004	0.81±0.004	1.17±0.01	1.08±0.01	0.26	**0.53**	***5.7E-28***	***7.4E-37***

Mean±se fold change between SD and SS or between SS and ZT0 of the involved genes is shown for each enriched function. Fold changes were compared between young and old animals using paired Wilcoxon Signed Rank, and P-values < 0.05 are shown in bold italic. Effect sizes were calculated as Cohen's d indicating standardized difference between two means (of young and old mice). Effect sizes >=0.5 (medium effect) is shown in bold. *genes from three *Signal/transmembrane* functional clusters enriched among common, young-specific, and old-specific sleep genes were combined and compared between the age groups.

Table S5. Comparison of the mean fold changes between young and old mice of the enriched functions among wake genes

source	Functions	Fold change between SD and SS		Effect size between young and old (d)	P.value
		Young	Old		
common	ERstress	1.46±0.05	1.4±0.03	0.22	***8.1E-05***
	Reg.transcription	1.48±0.02	1.44±0.03	0.13	***1.7E-13***
	Response to VEGF	1.84±0.25	1.7±0.21	0.20	***0.037***
	Dephosphorylation	1.54±0.1	1.51±0.09	0.04	0.601
	RhythmicProcess	1.54±0.07	1.51±0.07	0.07	0.249
	FoxO sigaling	1.62±0.08	1.55±0.09	0.16	0.091
	Response to cAMP	2.03±0.23	2.14±0.33	0.01	0.934
	MAPK signaling	1.69±0.08	1.62±0.07	0.13	***1.1E-05***
	Kinase activity	1.51±0.03	1.43±0.03	0.23	***1.8E-12***

	Cytokine activity	1.69±0.11	1.62±0.11	0.16	***0.007***
	Dioxygenase	1.55±0.06	1.47±0.05	0.31	***0.043***
	Fat cell differentiation	1.98±0.43	2.18±0.67	0.01	0.277
Y-specific	Actin bindng	1.32±0.02	1.14±0.01	**1.61**	***4.5E-13***
	GTPase activation	1.3±0.04	1.14±0.02	**1.18**	***3.0E-08***
O-specific	Lipid transport	1.14±0.01	1.2±0.02	**1.31**	***0.004***
	Regu.transcription	1.13±0.01	1.22±0.01	**0.96**	***2.9E-12***
	Tcell receptor	1.16±0.06	1.22±0.05	**0.60**	0.063

Mean±se fold change between SD and SS of the involved genes is shown for each enriched function. Fold changes were compared between young and old animals using paired Wilcoxon Signed Rank, and P-values < 0.05 are shown in bold italic. Effect sizes were calculated as Cohen's d indicating standardized difference between two means (of young and old mice). Effect sizes >=0.5 (medium effect) is shown in bold.

Table S7. expression changes of core clock genes between SS and SD and between young and old mice

	FDR				Average fold change					
	SD vs. SS		O vs. Y		SS vs. ZT0		SD vs. SS		O vs. Y	
**Gene**	**Young**	**Old**	**ZT0**	**SS**	**Young**	**Old**	**Young**	**Old**	**ZT0**	**SS**
Arntl	***6.5E-13***	***6.2E-14***	0.490	0.035	**0.83**	**0.79**	**1.25**	**1.31**	0.95	0.90
Clock	***4.3E-03***	0.101	0.354	0.994	1.00	0.89	1.12	1.13	1.11	0.99
Cry1	***1.3E-07***	0.070	0.442	***9.3E-05***	**0.80**	0.87	**1.15**	1.02	1.05	1.15
Cry2	***7.9E-03***	0.077	0.336	0.953	**0.85**	0.98	1.11	1.04	0.91	1.05
Per1	***2.2E-10***	***2.9E-09***	0.030	0.994	**0.46**	**0.80**	**1.87**	**1.70**	**0.61**	1.05
Per2	***2.3E-33***	***1.6E-26***	0.025	0.011	**0.58**	**0.82**	**1.98**	**1.69**	**0.81**	**1.15**
Per3	0.194	0.027	0.819	0.010	1.03	1.13	0.94	0.93	1.02	1.11
Dbp	***1.3E-20***	***1.1E-19***	0.276	0.613	**1.52**	**1.89**	**0.52**	**0.51**	**0.85**	1.05
Tef	***6.6E-12***	***7.0E-09***	0.673	0.730	1.02	0.90	0.89	0.90	1.02	1.03
Hlf	***3.4E-04***	0.055	0.051	0.777	0.93	0.93	**0.84**	0.90	**1.23**	1.07
Nr1d1	0.028	0.014	0.202	0.867	1.03	**1.43**	**0.83**	**0.76**	**0.75**	1.04
Nr1d2	***9.3E-03***	0.522	0.225	0.981	**1.16**	1.05	0.86	0.94	**1.17**	1.06

FDR values <0.01 for the comparisons between SD and SS or between old and young are shown in bold and italic. Fold changes < 0.85 or >1.15 (equivalent to absolute $\log_2$ fold change>0.2) are shown in bold.

Table S8. Expression changes of synaptic homeostasis related genes between SS and SD and between young and old mice

		FDR				Average fold change					
		SD vs. SS		O vs. Y		SS vs. ZT0		SD vs. SS		O vs. Y	
Function	Gene	Young	Old	ZT0	SS	Young	Old	Young	Old	ZT0	SS
Trans-cription factor	Fos	***4.9E-20***	***1.6E-21***	**0.046**	0.994	**0.08**	**0.13**	**4.72**	**4.86**	**0.62**	1.05
	Fosb	***1.1E-24***	***3.2E-23***	0.055	0.739	**0.27**	**0.46**	**3.25**	**2.88**	**0.70**	**1.19**
	Egr1	***3.2E-25***	***1.7E-27***	0.090	0.777	**0.29**	**0.37**	**2.60**	**2.77**	**0.74**	0.95
	Egr2	***6.4E-17***	***5.2E-18***	***2.7E-04***	0.714	**0.05**	**0.09**	**7.76**	**11.47**	**0.37**	**0.61**
	Arc	***3.0E-23***	***1.6E-22***	***0.006***	0.994	**0.08**	**0.17**	**7.40**	**7.07**	**0.40**	0.92
	Bdnf	***2.2E-26***	***4.3E-28***	0.255	0.109	**0.79**	**0.78**	**2.30**	**2.48**	**0.85**	**0.83**
	Ntrk2	***1.0E-07***	***5.0E-08***	**0.025**	0.994	1.14	1.00	1.10	1.14	1.14	1.01
Post-synaptic	Gria1	0.095	0.011	0.281	0.994	1.04	0.98	1.05	1.02	1.08	1.02
	Grin2a	0.198	0.026	0.965	0.937	1.09	1.05	1.06	1.03	1.01	0.97
	Gria4	***5.3E-06***	0.497	0.725	0.516	1.12	0.98	**0.82**	0.94	1.04	0.91
	Grin3a	***1.5E-09***	***1.7E-05***	0.744	***0.001***	1.13	0.98	**0.79**	**0.82**	0.97	**0.84**
	Cacna1b	***5.3E-07***	0.040	0.519	0.266	1.09	1.07	0.89	0.96	0.96	0.94
	Cacna2d1	***1.2E-06***	0.143	0.414	0.529	**1.21**	0.96	**0.73**	0.88	1.12	0.89
	Nlgn1	***4.7E-06***	***6.1E-04***	0.271	0.994	**1.25**	1.04	**0.79**	**0.84**	1.14	0.94
Pre-synaptic	Pclo	***1.5E-07***	0.889	0.131	0.994	**1.17**	0.91	**0.83**	1.02	**1.28**	0.99
	Syn1	***0.005***	0.179	0.334	**0.011**	1.09	0.95	0.91	0.99	1.07	0.93
	Syn2	***1.6E-05***	0.055	0.263	0.876	1.04	0.95	0.90	0.94	1.06	0.97
	Nrxn1	***1.2E-07***	***0.001***	0.233	0.994	**1.16**	1.00	**0.84**	0.87	1.11	0.96
	Nrxn3	***1.5E-07***	0.163	0.218	0.499	**1.19**	1.06	0.87	0.97	1.10	0.98
	Slit2	***8.6E-14***	***0.002***	0.137	***9.0E-10***	**1.22**	1.06	**0.73**	0.87	0.86	**0.75**
	Reln	***7.5E-10***	0.322	0.297	0.198	**1.22**	1.02	**0.82**	0.95	1.09	0.91
Others	Lsg1	***6.1E-09***	0.171	0.631	***0.009***	0.93	1.00	**1.19**	1.05	1.03	1.11
	Gfap	***1.4E-04***	0.389	0.379	***6.0E-04***	0.87	1.08	**1.44**	0.94	**1.20**	**1.49**

	Slc22a3	***7.6E-10***	0.579	0.893	***0.006***	**1.65**	0.96	**0.40**	0.91	1.05	**0.61**

FDR values <0.01 for the comparisons between SD and SS or between old and young are shown in bold and italic, and FDR <0.05 are shown in bold only. Fold changes < 0.85 or >1.15 (equivalent to absolute log_2 fold change>0.2) are shown in bold.
